# Possible stochastic sex determination in *Bursaphelenchus* nematodes

**DOI:** 10.1038/s41467-022-30173-2

**Published:** 2022-05-11

**Authors:** Ryoji Shinya, Simo Sun, Mehmet Dayi, Isheng Jason Tsai, Atsushi Miyama, Anthony Fu Chen, Koichi Hasegawa, Igor Antoshechkin, Taisei Kikuchi, Paul W. Sternberg

**Affiliations:** 1 School of Agriculture, Meiji University, Kawasaki, Japan; 2 Division of Parasitology, Faculty of Medicine, University of Miyazaki, Miyazaki, Japan; 3 Present Address: Forestry Vocational School, Duzce University, Duzce, Turkey; 4 Biodiversity Research Center, Academia Sinica, Taipei, Taiwan; 5 Division of Biology and Biological Engineering, California Institute of Technology, Pasadena, USA; 6 Department of Environmental Biology, College of Bioscience & Biotechnology, Chubu University, Kasugai, Japan

**Keywords:** Development, Evolutionary genetics, Comparative genomics

## Abstract

Sex determination mechanisms evolve surprisingly rapidly, yet little is known in the large nematode phylum other than for *Caenorhabditis elegans*, which relies on chromosomal XX-XO sex determination and a dosage compensation mechanism. Here we analyze by sex-specific genome sequencing and genetic analysis sex determination in two fungal feeding/plant-parasitic *Bursaphelenchus* nematodes and find that their sex differentiation is more likely triggered by random, epigenetic regulation than by more well-known mechanisms of chromosomal or environmental sex determination. There is no detectable difference in male and female chromosomes, nor any linkage to sexual phenotype. Moreover, the protein sets of these nematodes lack genes involved in X chromosome dosage counting or compensation. By contrast, our genetic screen for sex differentiation mutants identifies a *Bursaphelenchus* ortholog of *tra-1*, the major output of the *C. elegans* sex determination cascade. Nematode sex determination pathways might have evolved by “bottom-up” accretion from the most downstream regulator, *tra-1*.

## Abstract (web summary)

In most species, sex is determined by genetic or environmental factors. Here, the authors present evidence that sex determination in *Bursaphelenchus* nematodes is instead likely to be regulated by a random, epigenetic mechanism.

## Introduction

Sexual reproduction in animals results in powerful selective forces that optimize male and female phenotypes over the course of evolution. Since sexual dimorphism likely originated from the last common ancestor of celomate bilaterians, comprising most animals^1^, the mechanism that specifies sex is expected to be highly conserved; however, sex determination mechanisms are unexpectedly diverse and rapidly evolving (e.g., Dubendorfer et al.^2^; Bachtrog et al.^3^).

Sex-determination mechanisms have been broadly classified based on the type of switch that triggers the sex determination cascade. In the common “genetic sex determination (GSD)” sex is determined by a sex chromosome (e.g., *Sry* gene in mammals^4^ or the X:A ratio in the fruitfly *Drosophila melanogaster*^5^ and the nematode *Caenorhabditis elegans*^6,7^ or an autosomal gene or genes (e.g., polygenic in fish^8^, and a single-sex determination locus in house flies^9^). In “environmental sex determination (ESD)”, sex is determined by temperature (e.g., reptiles^10^) or local sex ratio of males to females (e.g., fish^11^). An often-overlooked sex determination mechanism is based on random factors. For example, in ciliate *Tetrahymena thermophila*, alleles at the *MAT* locus determine the distribution of probabilities with which one of seven mating types is expressed^12^. However, very little is known about how much randomness is actually involved in sex determination.

Sex determination takes place not only through the initial switch but also through a cascade of interacting genes. At the bottom of the cascade are the terminal regulators of sex determination, which integrate position and development information in sexual identity and coordinate male or female development. Despite the essential nature of sex determination, the genetic cascade of it is only understood in surprisingly few animals, such as the nematode *C. elegans*, the fly *D. melanogaster*^13^, and the mouse *Mus musculus*^14^.

Sex determination of *C. elegans* is one of the most well-known aspects of its biology. *C. elegans* has two sexes: self-fertilizing hermaphrodite and male. The hermaphrodite is a modified female whose fourth larval stage produces sperm to be used later to fertilize oocytes^15^. A *C. elegans* male is produced by nondisjunction of the X-chromosome. The initial switch for sex determination in *C. elegans* is the ratio of X chromosomes (X), to sets of autosomes (A)^6,7,16^. XX embryos develop as hermaphrodites and XO embryos as males. The set of sex chromosomes inherited determines the activity of the master regulator Transformer-1 (TRA-1), a zinc-finger transcription factor that directs sexual differentiation through acting on the intermediate factors in the sex determination cascade. High TRA-1 activity in XX animals promotes hermaphrodite (female) differentiation, whereas low TRA-1 activity in XO animals allows for male differentiation. However, there are still many questions regarding the diversity of sex determination mechanisms and their evolution among nematodes^17–19^. Many studied nematode species have an XX/XO system for sex determination in which females/hermaphrodites are XX and males are XO, like *C. elegans*, though some animal parasitic nematodes have an XX/XY mechanism^20,21^. Furthermore, Mermithidae and some plant-parasitic nematodes have ESD^22,23^, though the molecular mechanisms underlying ESD are totally unknown. In particular, a major issue that remains largely unsolved is how sex determination pathways evolved among nematodes.

Here, we focus on the sex determination of the nematode genus *Bursaphelenchus*, containing over 100 fungal-feeding and/or plant-parasitic species and two economically relevant plant pathogens: *B. xylophilus*, the pathogen of pine wilt disease, and *B. cocophilus*, the pathogen of red ring disease. We previously reported that *B. okinawaensis* has self-fertilizing hermaphrodites that spontaneously produces males with low frequency (< 1% of the population)^24^, similar to *C. elegans*. Strikingly, the sex ratio of progeny produced from a cross fertilization between a *B. okinawaensis* male and a *B. okinawaensis* hermaphrodite is still highly skewed (0.3% of males) towards hermaphrodites. If males have an XO karyotype, then this cross-mating should yield an approximately 1:1 ratio of males to hermaphrodites in the progeny. For example, in *C. elegans*, approximately 50% of progeny after cross-mating between a male and hermaphrodite are male. Moreover, it has been suggested that the sex determination of the closely-related species *B. xylophilus* is not an XX/XO system, based on DAPI staining cytology^25^. *B. xylophilus* is diecious and therefore always reproduces by mating. The sex ratio of *B. xylophilus* is generally biased towards females, but there is a certain variation in sex ratio, with the percentage of males varying between isolates in the range of 25–55%^26^.

Given the sex ratio distortion and cytological observation in the above studies, we hypothesize that *Bursaphelenchus* nematodes may have a strikingly distinct sex determination system from *C. elegans* in this work. To test this hypothesis, we investigate both the initial trigger for sex determination using a genome-wide comparative analysis, and the genetic cascade that follows that initial switch in *Bursaphelenchus* nematodes using forward genetic screening and genomics. We find that their sex differentiation is more likely triggered by random, epigenetic regulation than by more well-known mechanisms of chromosomal or ESD. Furthermore, this study shows the first sex determination gene in Clade IV nematodes and indicates the sex determination pathway of nematodes has arisen “bottom-up” during the nematode evolution.

## Results

### No genome difference between sexes in *B. xylophilus* and *B. okinawaensis*

**Fig. 1:**
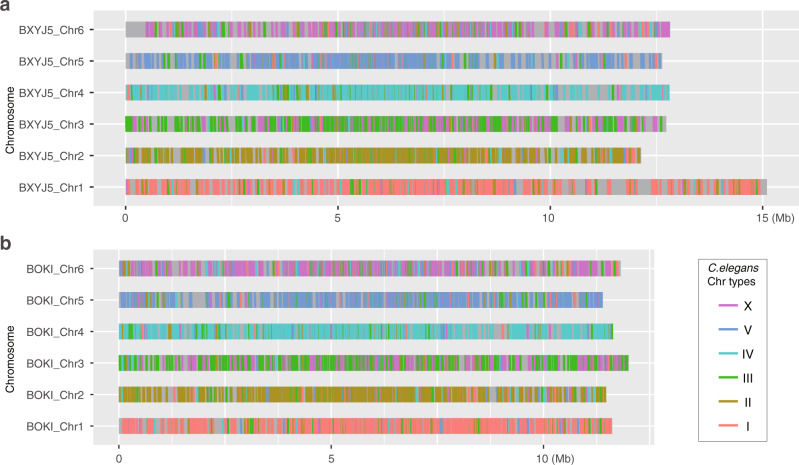
Schematic overview of the six chromosomes of *B. xylophilus* and *B. okinawaensis*. **a** *B. xylophilus*. **b** *B. okinawaensis*. One-to-one orthologs with *C. elegans* genes were used to visualize syntenic relationships.

**Fig. 2:**
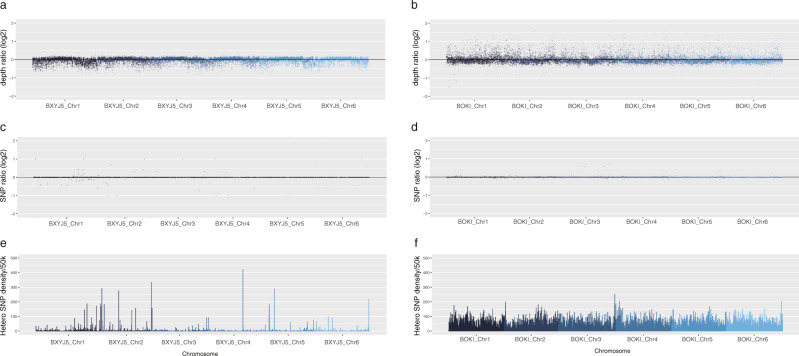
Sex-biased read-depth and SNP density along the six chromosomes. Depth of coverage was calculated by a 5 kb sliding window and centered by median depth of whole genome. Log2 ratio of read-depth of male sequencing data relative to female/hermaphrodite is plotted (*y* axis) across genomic regions (*x* axis) for *B. xylophilus* (**a**) and *B. okinawaensis* (**b**). Heterozygous SNP density was calculated with 50 kb windows for *B. xylophilus* female (**e**) and *B. okinawaensis* hermaphrodite (**f**). Log2 ratio of SNP numbers in male relative to female/hermaphrodite are plotted (*y* axis) across genomic regions (*x* axis) for *B. xylophilus* (**c**) and *B. okinawaensis* (**d**).

The karyotype of *B. okinawaensis* indicates that both hermaphrodites and males have six pairs of bivalents during Diakinesis (Supplementary Fig. 1a–f). Since the sex ratio of *B. okinawaensis* is highly biased toward hermaphrodite even after cross-mating, it was difficult to observe the karyotype in the zygote male after a cross. Therefore, we investigated potential genetic differences between hermaphrodites and males by genome sequencing. Since there was no available genome information for *B. okinawaensis*, de novo sequencing was carried out on the genomic DNA extracted from mixed-stage nematodes including eggs, larvae, and adults. We previously reported a draft genome of *B. xylophilus*^27^ but used new sequencing technologies to obtain higher contiguity. We assembled their genomes into six chromosomes comprising assemblies of 70.0 Mb in *B. okinawaensis*^28^ and 78.3 Mb in *B. xylophilus*^29^ (Supplementary Table S1). We found conserved macrosynteny between *Bursaphelenchus* and *C. elegans* in four chromosomes (corresponding to *C. elegans* chromosomes *I*, *II*, *IV*, and *V*), but extensive rearrangement between the other two chromosomes (corresponding to *C. elegans* chromosome *III* and *X*; Fig. 1). Sex-specific short reads obtained from each sex stage of *B. okinawaensis* and *B. xylophilus* (Supplementary Table S2) were mapped onto the genome assemblies and the depth of coverage and SNP density were calculated. Depth of coverage per 5-kb window showed a similar density distribution in all chromosomes regardless of sex (Fig S2) and the log2 ratios of the two sexes are between −0.5 and 0.5 for >99% genomic regions (Fig. 2). Nor was there any elevated SNP density per 50-kb window identified in either of the sexes. Haplotypic K-mer analysis of the sex-specific short reads using Smudgeplots^30^ indicated that both *B. xylophilus* and *B. okinawaensis* are diploid, with a diploid (AB) confidence of 0.95 and 0.87 for *B. xylophilus* and 1.0 for *B. okinawaensis* samples (Fig, S1g, h, i, j). Some short fragments were detected as possible Y chromosome-specific sequences by the K-mer-based Y chromosome detection method^31^ (Table S3), but those fragments were mostly A-T homopolymers. Therefore, the two sexes of *B. okinawaensis* and *B. xylophilus* are likely to have essentially identical genomes, and thus no sex chromosomes.

### Sex-determination locus mapping in *B. xylophilus*

If a sex determination locus exists but is not detectable from genome analysis, it should nonetheless be detected by genetic linkage. *B. xylophilus* reproduces gonochoristically and there is a higher possibility that females or males have a sex determination locus on the chromosomes than the hermaphroditic species *B. okinawaensis*, which produces male and hermaphrodite progeny through self-fertilization at a constant rate over many generations. We used five SNP markers for the two *B. xylophilus* strains (Ka4C1 and S10-P9) for each of the six chromosomes (Supplementary Fig. 3; Supplementary Table 4) and genotyped F1 cross progeny (Supplementary Figs. 4, 5). If there is a region linked to either the male or female phenotype, it is expected that the band pattern between males and females would be different for the specific markers near the sex-determining locus. However, we observed no significant difference in any of the markers, indicating that *B. xylophilus* does not have a chromosomal sex-determining locus.

### Effect of environmental stimuli and developmental history on sex determination of *B. okinawaensis*

To test the possibility that *B. okinawaensis* utilizes ESD, we examined the effect of various environmental stimuli and developmental histories on their sex determination (Supplementary Table 5). Heat shock (40 °C, 4 h) and incubation for 7 days at 30 °C induced a relatively higher ratio of males compared to normal growing conditions; however, the male ratio was still always lower than 2%. When L4 worms were exposed to high temperatures (40 °C) and incubated at 30 °C, the male ratio was increased from 0.49% to 1.40% and 1.70%, respectively. When worms were cultured at a high population density and exposed to 10% ethanol, the male ratio was 0.51% and 0.15%, respectively, and there was no increase in the male ratio. Thus, the effect of the environmental stimuli was limited. By contrast, passage through the dauer stage decreased the number of male progeny. Specifically, only hermaphrodites were observed among 2639 F1 progeny after dauer recovery. In the F2 generation after dauer recovery, the male ratio was 0.79, with no significant difference compared to the control. The number of nematodes examined was limited because it was difficult to induce recovery from the dauer stage in *B. okinawaensis*, even when the larvae were placed in food-rich conditions. No effect was observed by L4 starvation on the sex ratio.

### Lack of symbiotic microbes in *B. okinawaensis*

We also investigated whether *Bursaphelenchus* nematodes have symbiotic microbes. In insects, symbiotic microorganisms such as *Wolbachia* are able to manipulate their host sex determination system^32^. We examined if the sex ratio can be changed by treatment of antibiotics and found no significant change in the sex ratio (Supplementary Fig. 6a). We next attempted to detect bacteria by PCR with bacteria-specific primers (Supplementary Table 6), and no symbiotic bacteria were detected (Supplementary Fig. 6b). Furthermore, no clear non-nematode genetic material was observed in the genomic DNA sequence. The sex determination of these nematodes is thus likely not manipulated by symbiotic microorganisms.

### Conservation of sex determination and dosage compensation pathway genes in *B. xylophilus* and *B. okinawaensis*

**Fig. 3:**
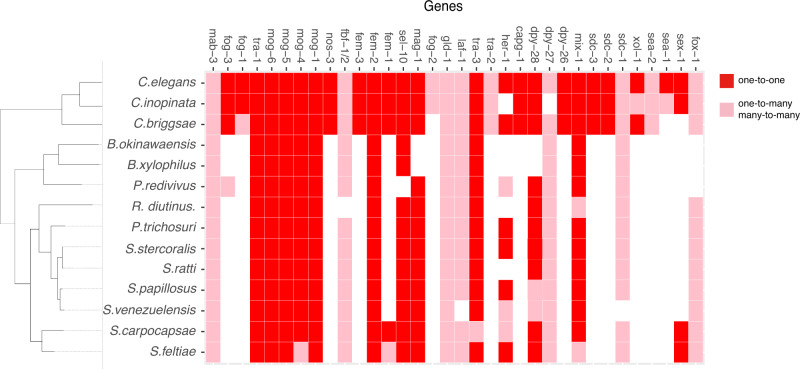
Identified sex determination genes in Bursaphelenchus and other clade IV nematodes. Orthology of genes involved in the sex determination and dosage compensation in *C. elegans* was predicted using OrthoFinder for *B. xylophilus*, *B. okinawaensis*, and two *Caenorhabditis* species (*Caenorhabditis inopinata* and *Caenorhabditis briggsae*) and clade IV nematodes (*Panagrellus redivivus*, *Rhabditophanes* sp. KR3021, *Parastrongyloides trichosuri*, *Strongyloides papillosus*, *Strongyloides ratti*, *Strongyloides stercoralis*, *Strongyloides venezuelensis*, *Steinernema carpocapsae,* and *Steinernema feltiae*).

We identified *B. xylophilus* and *B. okinawaensis* orthologs of *C. elegans* sex determination genes (Fig. 3). Widely conserved sex determination genes are also conserved in *Bursaphelenchus*, including the terminal regulator *tra-1*, which controls all aspects of somatic sexual differentiation, *tra-3*, and *fem-2*. However, orthologs of over half of *C. elegans* sex determination genes, especially genes involved in the upstream of the pathway, were not present, e.g., the X-chromosome counting factors *fox-1*/*sex-1* and the *C. elegans* primary sex determination switch gene *xol-1*. Also, the *xol-1* targets SDC proteins are also likely absent in *B. xylophilus* and *B. okinawaensis*. Some genes comprising the dosage compensation protein complex (*dpy-26*/*28*), which are recruited by SDC, were also not present in *Bursaphelenchus*. The absence of these genes is consistent with our hypothesis that *B. xylophilus* and *B. okinawaensis* are not dependent on XO sex-determination system.

In *C. elegans*, signal transduction from SDC to *tra-1* contains several genes including *her-1*, *tra-2/3*, *sel-10,* and those encoding the FEM proteins. Among those *tra-1* upstream genes, we identified only orthologs of *tra-3, fem-2,* and *sel-10*, suggesting that *Bursaphelenchus tra-1* regulation has highly diverged. Other genes conserved in *Bursaphelenchus* include those having pleiotropic roles such as *mog-1, 4/5* all encoding pre-mRNA splicing factors, and *mog-6* encoding a nuclear cyclophilin^33^.

This conservation pattern is also observed in other Clade IV nematodes (Fig. 3). Although *tra-1* is well conserved in all the nematodes analyzed, most *tra-1* upstream genes are not identified in those nematodes, consistent with *Bursaphelenchus* (Supplementary Fig. 7).

### F2 chemical mutagenesis screening and characterization of mutants in *B. okinawaensis*

**Fig. 4:**
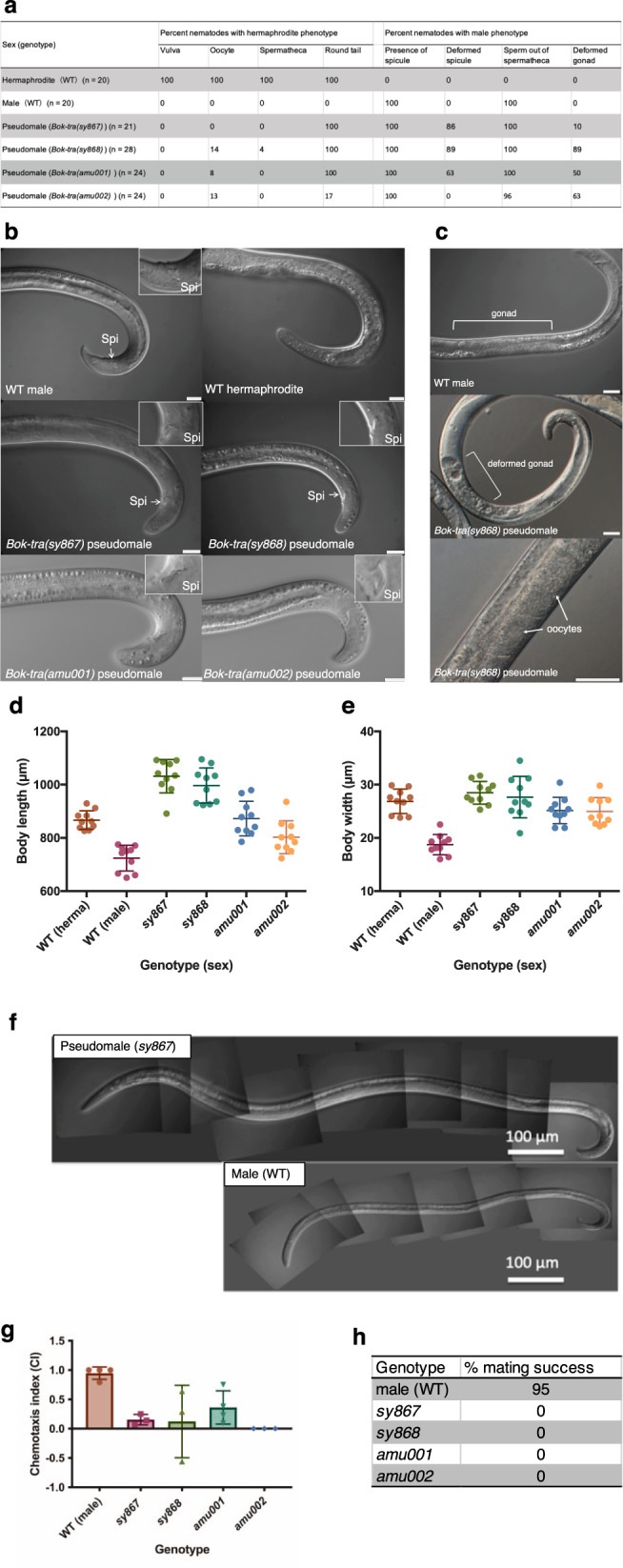
Characterization of sex determination mutants in *B. okinawaensis*. Summary of morphological phenotype in *B. okinawaensis* sex-determination mutants (**a**). Phenotypes were scored by DIC microscopy. Phenotypes of the tale shapes in wild-type (WT) and mutant pseudomales (**b**). Scale bar represents 20 μm. Spi: spicule. Phenotypes of the germline in WT male and mutant pseudomales (**c**). Scale bar represents 20 μm. Body length (**d**) and width (**e**) in WT and mutant pseudomales. Source data are available for **d**, **e** in the Source Data File. Images of ten distinct individuals for each genotype and sex were obtained by DIC microscopy and measured using ImageJ (ver. 1.53). Data are presented as mean values ± standard deviation. Whole-body of WT male and pseudomale of *Bok-tra(sy867)* (**f**). Chemotaxis on WT male and pseudomales in *B. okinawaensis* to the chemical cue of virgin females of *B. xylophilus* (**g**; source data are available in the Source Data File). The assay was performed according to Shinya et al.^78^ Chemotaxis index (CI) = ([number of nematodes at the test cue zone] − [number of nematodes at the control zone])/([number of nematodes at the test cue zone] + [number of nematodes at the control zone]). Chemotaxis assays were repeated four times for *Bok-tra(amu001)* and WT, and three times for others. Data are presented as mean values ± standard deviation. Percentages of mating success in WT male and pseudomales in *B. okinawaensis* with sperm-depleted old hermaphrodite of *B. okinawaensis* SH1 strain (**h**). Mating plates were prepared by placing a single young adult male of each genotype with two sperm-depleted old hermaphrodites on 1/10 MEA-chloramphenicol plates with yeast lawns. These plates were incubated at 25 °C; 48 h later, the number of progeny produced was counted. For each genotype, 20 biological replicates were examined.

To identify genes involved in the sex determination cascade of *B. okinawaensis*, we screened for EMS-induced mutants that produce males at a significantly higher rate in the F2 population. A total of four mutant lines—*Bok-tra(sy867)*, *Bok-tra(sy868), Bok-tra(amu001),* and *Bok-tra(amu002)*—were isolated through a screen of 43,176 mutagenized gametes (Fig. 4a). In each mutant, approximately two-thirds of hermaphrodites produce males at a higher ratio (18–25%), consistent with Mendelian segregation of recessive alleles. In addition, these males are morphologically abnormal (Fig. 4a, b, c). In mutants, adult male body length and width are both larger than in wild-type males (Fig. 4d, e). We characterized all mutants by observation under DIC optics and found that pseudomales of these mutants have an intersexual phenotype (Fig. 4). The shape of the tail in these mutant males is rounded like in hermaphrodites with abnormal copulatory spicules (Fig. 4b). Moreover, they have an abnormal gonad and only partially developed germline (Fig. 4c). Although *B. okinawaensis* males and hermaphrodites both have one-armed gonads and the gonad structure is grossly indistinguishable, 90% of *Bok-tra-1(sy867)* pseudomales had fully extended gonad containing sperm and none had oocytes. The number of sperm in the gonad in *Bok-tra-1(sy867)* pseudomales is well above the number made by a wild-type male. The gonad is not fully extended towards the anterior and apoptosis was often observed in the gonad. Mutant males were neither able to produce progeny nor be attracted to sex pheromone (Fig. 4g, h).

### Four transformer mutants define a single locus in *B. okinawaensis*

Complementation tests and linkage mapping of the four recessive sex determination mutations (*sy867*, *sy868*, *amu001,* and *amu002*) indicated allelism. Specifically, in each *inter se* heterozygous combination, cross-fertilized progeny still expressed a mutant male phenotype in the F2 population, indicating failure to complement. To identify SNPs for mapping, we obtained the genome sequence of the SH3 strain of *B. okinawaensis*, isolated from the Iriomote islands, and detected 0.11% SNPs (0.13% variants) between the SH1 and SH3 strains. Two SNP primers were synthesized for each of the six chromosomes (Supplementary Fig. 8; Supplementary Table 7) to distinguish between genotypes of SH1 and SH3. Mapping of *Bok-tra(sy867)* and *Bok-tra(sy868)* using 12 SNP markers indicated linkage to chr3 in both mutants (Supplementary Fig. 9). These results indicate the responsible gene causing this masculinization phenotype, *Bok-tra-1*, is located on chr3.

### Tra mutations affect *Bok tra-1a*

**Fig. 5:**
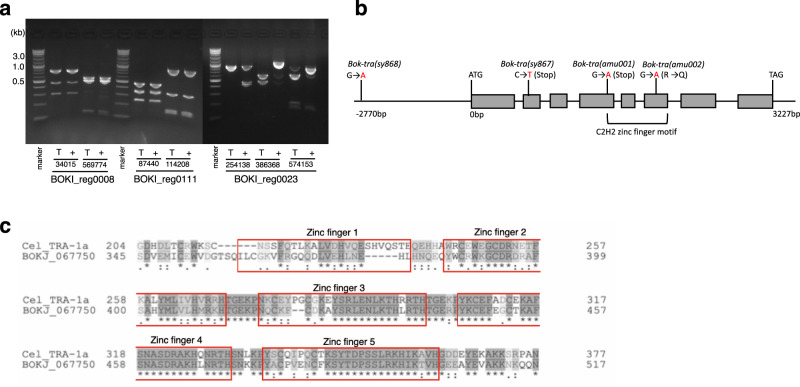
Molecular characterization of mutations in *B. okinawaensis*. SNP mapping for *Bok-tra(sy867)* (**a**). Each pair of lanes shows results from the SNP for each primer pair located in three regions (reg0008 on chr2, 0023 on chr3, and 0111 on chr5), using either the adult pseudomale of Tra (T) or adult hermaphrodite of wild-type (+) DNA template. Linkage is visible as an increase in the proportion of SH1 DNA in Tra lanes compared to the wild-type lanes, and is visible on BOKI.reg0023 in all primer sets. Gene structure of *Bok-tra-1*(BOKJ_0677500) and location of *Bok-tra-1* mutations (**b**). The conservation of the zinc-finger domain in TRA-1 between *C. elegans* and *B. okinawaensis* (**c**). The five zinc fingers are enclosed and numbered.

To identify the mutation in the *Bok-tra(sy867)* and *Bok-tra(sy868)* mutants, we called variants based on next-generation sequencing (NGS). 35.1 million and 37.4 million short reads were obtained from homozygous mutant males (*sy867*/ *sy867*) and wild-type hermaphrodites (+/+), respectively, and detected 511 unique variants. For *Bok-tra(sy868)*, 39.5 million and 37.8 million short reads were obtained from homozygous mutant males (*sy868*/ *sy868*) and wild-type hermaphrodites (+/+), respectively, and 454 unique variants were detected. Three genes, namely BOKJ0375500 (chr2), BOKJ0677500 (chr3), and BOKJ1395600 (chr5), had variants in both mutants. To confirm this prediction, SNP markers were designed near these three candidates, and used to examine linkage with the *Bok-tra(sy867)* allele (Supplementary Table 8). Only the markers on chr3 (Bok_reg0023) showed strong linkage (Fig. 5a). Therefore, BOKJ_0677500 is the causative gene for the hermaphrodite to pseudomale transformation. *Bok-tra(sy867)* results in a nonsense codon in the second exon prior to the zinc-finger domain (Fig. 5b). In *Bok-tra(sy868)*, a single G to A nucleotide substitution was detected in the region 2770 bp upstream of the start codon, and no mutation was found in the exons. We performed PCR and Sanger sequencing to identify the mutations in *Bok-tra(amu001)* and *Bok-tra(amu002)* using primers shown in Supplementary Table 9*. Bok-tra(amu001)* is a nonsense mutation occurring in the fourth exon. *Bok-tra(amu002)* has a single nucleotide substitution (G to A) in the sixth exon resulting in an amino acid substitution from R(CGG) to Q(CAG) at codon 495.

BOKJ_0677500 is a protein with five zinc fingers and is a homolog of *C. elegans tra-1a* (Fig. 5b, c). Sequence alignment analysis revealed high similarity in the zinc-finger domain sequence, whereas the other regions were highly divergent in sequence from *C. elegans tra-1* (amino acid identity = 14.0%) (Fig. 5c; Supplementary Fig. 7). OrthoFinder analysis also identified another ortholog (BOKJ_0684900) from *B. okinawaensis*. BOKJ_0684900 is much shorter (273 aa) than BOKJ_0677500 (717 aa) or *C. elegans tra-1a* (1110 aa), but still contains multiple zinc-finger domains. *C. elegans tra-1* has a short isoform *tra-1b* encoding TRA-1 (288 aa), which contains only the amino terminus and first two zinc fingers. While the function of *tra-1b* is unclear, it is possible that the *Bursaphelenchus* short orthologs have similar roles as *tra-1b*. Indeed, BOKJ_0684900 (*tra-1b*) has a similar expression pattern as the larger *tra-1* ortholog (BOKJ_0677500; *tra-1a*) in the RNA-seq analysis discussed below.

### RNA-seq analysis of *Bok-tra(sy867)* mutants

To examine genome-wide gene expression changes in a *tra-1* mutant, we obtained sex-specific RNA-seq data from the wild-type and *Bok-tra(sy867)* animals (Supplementary Table 2). We first compared expression of the wild-type males and hermaphrodites with a threshold of FDR 0.01 and fold-change >2 and categorized the genes into masculine genes (1922 genes upregulated in male), feminine genes (889 genes upregulated in hermaphrodite), and non-sex-biased genes (12089 genes) (Supplementary Fig. 10, Supplementary Data 1). The masculine genes are enriched with GO terms kinase activity and phosphatase activity, whereas the feminine genes are enriched with terms structural constituent of cuticle, DNA replication, multicellular organismal process, and negative regulation of translation (Supplementary Data 2).

**Fig. 6:**
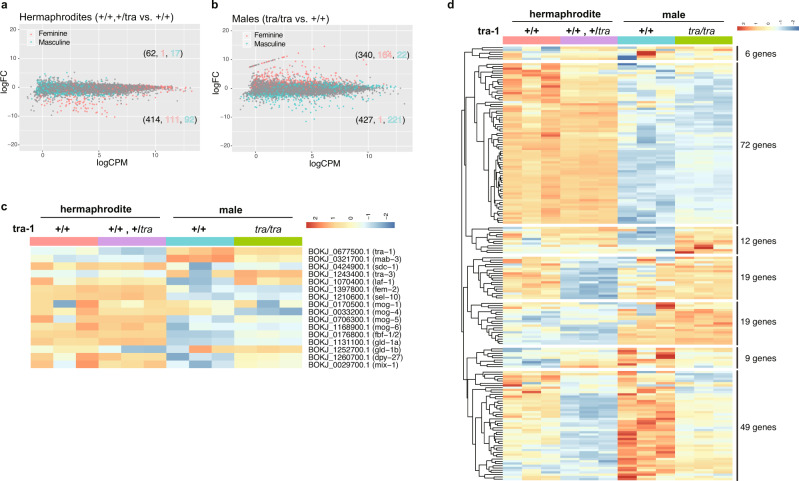
Gene expression in the *Bok-tra(sy867)* mutant. Summary of differential gene expression in the *tra-1* mutant compared to wild-type for *B. okinawaensis* hermaphrodites (**a**) and males (**b**). LogCPM and logFC represent the logarithm of counts per million reads and the log-transformed fold change in gene expression, respectively. Each dot represents the expression of one gene. The numbers of upregulated and downregulated genes in the *tra-1* mutant are shown in parenthesis. Feminine and masculine genes detected by a comparison of wild-type hermaphrodite and male (see Supplementary Fig. 10) were shown in red and blue, respectively. **c** Gene expression of *B. okinawaensis* orthologues of genes involved in sex determination and dosage compensation (see Fig. 3). **d** Gene expression of *B. okinawaensis* orthologues of genes associated with TRA-1 proteins in *C. elegans*.

A comparison of wild-type (*+/+*) and the *tra-1* mutant population (a mixture of two-thirds of *tra*/*+* and one-third of +*/+*) hermaphrodites detected 62 upregulated and 414 downregulated genes in the *tra-1* mutant (Fig. 6a, Supplementary Data 3, 4). In this comparison, a similar number of feminine genes (111 genes) and masculine genes (92 genes) were downregulated, but genes with high-fold changes (fold-change >4) were dominated by feminine genes (66/103 genes).

By contrast, in the male comparison (+/+ males vs *tra*/*tra* pseudomales), we detected 164 upregulated feminine genes and 221 downregulated masculine genes (221 genes; Fig. 6b, Supplementary Data 5, 6). There were fewer DE genes and smaller fold changes in this male-pseudomale comparison than in the wild-type male and female comparison. Though *Bok-tra(sy867)* is likely null, the effect of *Bok-tra-1a* on somatic sex determination is incomplete, suggesting additional factors might specify female development in *Bursaphelenchus*. TRA-1-independent sex-determining genes are likely to be among these genes differentially expressed between +/+ males and *tra*/*tra* pseudomales. Among the genes with a >5-fold change, there are many structural proteins, e.g., cuticlin, collagen, although no orthologs for genes known to be involved in sex determination in *C. elegans*. The body length and width of pseudomales are almost same as hermaphrodites and significantly longer than those of wild-type males. The structural proteins upregulated in *Bok-tra(sy867)* mutant might be involved in these differences in body size, and these are regulated in a *tra-1* independent manner. Half of genes with a >5-fold-change (59 /121 genes) are of unknown function.

We next examined expression patterns of orthologs of the *C. elegans tra-1* pathway and found that the expression level of *tra-1* itself was higher in wild-type males than hermaphrodites and even higher in pseudomales. *tra-3* and *mab-3* showed similar expression patterns in the male/pseudomale comparison (Fig. 6c). These genes are likely to be *tra-1-*dependent and involved in sex determination in *Bursaphelenchus* and might be ancestral sex-determining genes in nematodes. Given that TRA-1 activity is considered to be low in wild-type males as in the *tra*/*tra* mutants based on the information in *C. elegans*, the DE genes in the male/pseudomale comparison primarily contained genes regulated by sex determination pathways independent from the *tra-1a* gene. Genes downregulated commonly in the wild-type male (*+*/*+*) and mutant male (*tra*/*tra*) but not in the hermaphrodites (*+*/*+* or *tra/+*) contain the genes potentially involved in feminization (Fig. 6c; Supplemental Fig. 10); this group includes orthologs of *C. elegans* sex determination genes, e.g., BOKJ_1397800.1 (*fem-2*), BOKJ_0176800.1 (*fbf-1,−2*), BOKJ_1131100.1 (*gld-1a*), BOKJ_ 1260700.1 (*dpy-27*), BOKJ_0029700.1 (*mix-1*), BOKJ_0706300.1 (*mog-5*) and BOKJ_1210600.1 (*sel-10*). Although core somatic sex determination genes are also required for germline sex determination in *C. elegans*^34^, these two processes differ in fundamental ways to allow each sex to specify the appropriate germ cell fate at the appropriate time; we compared gene expression profiles between males and hermaphrodites having both somatic and germline tissue, and are thus unable to distinguish somatic or germline expression.

We also investigated potential *Bok-tra-1* target genes involved in male-specific development. In *C. elegans* 384 genes associated with TRA-1 were identified by ChIP-seq^35^. Hierarchical clustering grouped the expression patterns of the 168 *B. okinawaensis* orthologs of these genes into seven clusters (Fig. 6d). We found gene groups commonly upregulated (cluster 2: 72 genes; Supplementary Data 7) and downregulated (cluster 5: 19 genes; Supplementary Data 8) in the wild-type male (*+/+*) and mutant male (*tra*/*tra*) compared to the hermaphrodites (Fig. 6d). In *C. elegans*, some genes are known to be involved in male-specific development, e.g*., mab-3* (inhibitor of yolk production). *mab-3* has at least two distinct functions in male development: inhibition of yolk protein production by males, and specification of male sense organ differentiation. *Bok-mab-3a* (BOKJ_0321700) were upregulated in both wild-type and mutant males but not in hermaphrodites, suggesting that *Bok-mab-3* is targeted by *Bok-tra-1* and regulates a subset of sex-specific male development. The *tra-1* downstream pathway is thus relatively highly conserved compared to the upstream part of the pathway between *Bursaphelenchus* and *Caenorhabditis*.

## Discussion

There is no difference in the number of chromosomes between males and females in *B. xylophilus*^25^ and between males and hermaphrodites in *B. okinawaensis* (Fig. 1; Supplementary Fig. 1). However, it was still unclear whether all chromosomes are morphologically identical. Therefore, we established chromosome level genome assemblies using Nanopore long sequencing and HiC analyses and performed a genome comparison between males and females/hermaphrodites. No clear differences were identified between males and hermaphrodites/females in the genome coverage at 5 kb window and SNP density at 10 kb window in *B. okinawaensis* and *B. xylophilus*. There is still a possibility that small differences between the two sexes can arise from the gap region of the genome, but it is unlikely because the hermaphrodites of *B. okinawaensis* stably produce males during self-fertilization. We conclude that *B. okinawaensis* and *B. xylophilus* use a non-sex chromosomal mechanism for their sex determination.

In the gonochoristic *B. xylophilus*, sex conceivably could be determined by an autosomal sex-determining locus similar to the case in some populations of the housefly *Musca domestica*^2^, though this is unlikely in the hermaphroditic *B. okinawaensis* because the hermaphrodite produces males and hermaphrodites through self-fertilization at a constant rate over many generations and the proportion of males did not increase even after repeated mating with males. To test for the presence of autosomal sex-determining loci in *B. xylophilus*, association analysis using SNP markers was carried out using two distinct strains, and we failed to detect linkage of sex and any chromosomal region. The results of genome analysis and this association analysis indicate that the sex determination of *Bursaphelenchus* nematodes is not genetically determined.

After ruling out chromosomal sex determination, we investigated the possibility of ESD, in which environmental factors such as temperature, nutrient availability, and photoperiod have been shown to affect sex determination^10,36,37^. We found only small effects of high temperature, starvation, or passage through dauer larvae. Worms passaged through dauer stage (diapause) did not develop into males. The appearance of males after dauer recovery was thus suppressed by unknown epigenetic mechanisms. The experience of dauer stage can affect the sex of nematodes in *Auanema rhodensis* (*aka Rhabditis* SB347)^38^. The mechanism by which epigenetic changes in post-dauer stage affect sex determination might be widely conserved among nematodes that reproduce hermaphroditically. However, it is difficult to investigate this mechanism because we cannot distinguish between male and hermaphrodite at the L2 stage of *B. okinawaensis* at present. Under strict ESD systems, sex ratio is predicted to be a stepwise function of environment^39^. Throughout our experiments, the male ratio was always lower than 2% regardless of environmental stimuli, inconsistent with ESD. Even within the host tree, the sex ratio does not change significantly^26^. In *C. elegans*, sex is determined by the ratio of autosomes to sex chromosomes but the male ratio can be increased by heat shock, through sex chromosome nondisjunction^16^. Since our genome analysis clearly showed that *B. okinawaensis* has no genetic sex-determination system, the male ratio would have to be increased by a different mechanism. Considering that the offspring produced through self-fertilization in *B. okinawaensis* are essentially isogenic clones, it is clear that genetic differences are not required for sex determination in *B. okinawaensis*. In addition, even under fixed environmental conditions, genetically identical individuals of *B. okinawaensis* differentiate into hermaphrodites and males. We infer that the sex of *B. okinawaensis* nematodes is mainly determined by stochastic expression of an unknown trigger gene and/or developmental noise, though limited epigenetic control associated with dauer formation was observed. In other words, sex differentiation occurs as a result of random events during development.

Clonal populations of organisms often show substantial phenotypic variation. Such heterogeneity is essential for many aspects of biological processes and can occur by stochasticity or noise in gene expression. In particular, this has been well-documented in by microbiologists. A notable example is the formation of persister cells in bacteria^40^. Persisters are dormant or slow-growing cells that are highly antibiotic-tolerant and usually compose a small fraction of bacterial populations. It is known that persisters are formed through a combination of stochastic and deterministic events. In multicellular organisms, it is relatively rare, but Perrin^39^ suggested that stochastic sex determination systems are as important as the genetic and ESD systems. Generally, it is difficult to prove the influence of developmental noise or the random factor in organisms having separate sexes (gonochoric) due to their heterogeneous genetic background. However, *B. okinawaensis* is a hermaphroditic species, and continuous self-fertilization results in isogenic clones. In this study, genetically identical individuals of *B. okinawaensis* became both hermaphrodites and males under fixed environmental conditions. This suggests that stochasticity plays a key role in their sex determination. Since we have shown that *B. okinawaensis* is genetically tractable, it might be a good model to test whether there is indeed random sex determination in metazoa. However, it remains difficult to completely rule out the possibility of ESD, or to rule in random sex determination by identifying its molecular mechanism.

Organisms use various strategies to cope with fluctuating environmental conditions. If there is a reliable environmental cue and the environment is predictable, conditional regulation of sex determination (i.e., ESD) should have an advantage for the fitness of the organism. However, under unpredictable conditions, the stochastic regulation of sex determination can be a bet-hedging strategy, a risk-spreading strategy that can be displayed by isogenic populations in unpredictably changing environments^41^. In diversified bet-hedging, a single genotype exhibits phenotypic heterogeneity with the expectation that some individuals will survive transient selective pressures. So far, *B. okinawaensis* has been isolated only from Ishigaki and Iriomote Islands. Since their vector beetle *M. maruokai* is endemic to these two islands and *B. okinawaensis* have a close relationship with this beetle, *B. okinawaensis* is probably endemic to Ishigaki and Iriomote Islands. These two islands have a subtropical marine climate with a continually warm temperature and high humidity. Although most aspects of *B. okinawaensis* ecology are still unclear, *B. okinawaensis* probably harbors in dead wood, where niche space and food are limited. Such an environment is likely to be rapidly changing and unpredictable^42^. Under this condition, the bet-hedging strategy of sex determination of *B. okinawaensis* could be more adaptive than genetic and ESD.

Using nearly-complete genome references of the two *Bursaphelenchus* species, we identified orthologs of *C. elegans tra-1*, *tra-3*, and *fem-2*, which are involved in the major sex determination pathway. Since the sequence of sex determination genes evolve rapidly, there is still the possibility that there are orthologs undetected in this study. All these ortholog genes identified are downstream genes in the sex determination cascade in *C. elegans*, and no upstream genes were present in *B. okinawaensis* and *B. xylophilus*. The fact that upstream genes like the X-chromosome counting factors *fox-1*/*sex-1*, initial trigger gene *xol-1,* and the dosage compensation genes *sdc-1,2,3, dpy-26*, *27*, and *28* were not found as one-to-one orthologs supports our hypothesis that *Bursaphelenchus* nematodes have a new sex determination trigger other than XX/XO system. In insects, the conservation of downstream genes of the sex determination gene cascade is higher than upstream genes including the trigger^43^. The evolution of a sex determination pathway seems to be similar among a wide range of organisms.

A random chemical mutagenesis screen recovered four sex-determination mutants. We cloned and identified these mutations by chromosome mapping with SNP markers and sequencing. The four mutations are in one of the two *B. okinawaensis* homologs of *C. elegans tra-1* (BOKJ_0677500). This gene, which we are naming *Bok*-*tra-1a*, has a similar function to the *C. elegans tra-1* isoform *tra-1a*. We name the other *tra-1* homolog, *Bok-tra-1b*. The mutations cause the development of pseudomales with an intersexual phenotype, with partial masculinization of the soma (displaying abnormal but male tails) but apparently complete masculinization of the germline. The phenotype of *Bok-tra-1(sy867)* animals is remarkably different from those of *C. elegans* XX males lacking *tra-1* function, which exhibit complete transformation to a male phenotype, and their nongonadal morphology is indistinguishable from that of wild-type of XO males. Some *C. elegans tra-1(lf)* XX males can sire progeny^44,45^. *Bok-tra-1* mutants exhibit complete transformation to a male phenotype in germline but only an incomplete transformation of somatic sex phenotype. This difference provides some insights into the mechanisms of sex determination of *Bursaphelenchus*. First, while *C. elegans tra-1* is the sole terminal regulator of somatic sex determination, additional factors might participate in dictating male versus female pathways in *Bursaphelenchus*. Second, the mosaic intersexual phenotypes suggest that sexual differentiation is cell autonomous in *Bursaphelenchus*. One of the four *Bok-tra-1* mutations (*sy868*) is 2770 bp region upstream of the start codon, potentially in a positively-acting regulatory element. In this study, no other sex-determination mutants were isolated other than the mutants with defect in *Bok-tra-1*. A few strains which produce males at a higher rate were isolated, but the male ratio was unstable during successive self-fertilization and did not show regular Mendelian segregation patterns. These unstable lines might be generated by a defect in the genes located upstream in the sex determination cascade. So far, *tra-1* has been functionally identified only in three nematodes: *C. elegans*, *C. briggsae*^46^, and *P. pacificus*^47^. All of these nematodes are classified as Clade V^48^ and most of the sex determination pathway genes are conserved among these species. On the other hand, *B. okinawaensis* is in Clade IV, whose genomes lack orthologs of most *C. elegans* sex determination genes; these nematodes are thought to have a completely different sex determination mechanism. For example, the root-knot nematodes, *Meloidogyne* spp., and cyst nematodes, *Heterodera* spp./*Globodera* spp. have been suggested to have ESD^49–51^. However, while it has been considered that sex is determined by the nutrient status of the host or under stress conditions in these nematodes, the molecular sex determination mechanisms are unknown. Our clear demonstration that the function of *tra-1* is conserved even in Clade IV nematodes will be useful for understanding the sex determination mechanisms of this group of worms, which includes many economically relevant parasitic nematodes. In the present study, along with Shinya et al.^24^, we establish methods for forward genetics in *B. okinawaensis*.

Our RNA-seq analysis of both sexes of wild-type and *tra-1* mutants revealed two key features of the potential role of *Bok-tra-1* in sex determination. First, orthologs of genes regulated by TRA-1 in *C. elegans* are similarly regulated by TRA-1 in *B. okinawaensis*. Notably, *mab-3*, an inhibitor of yolk synthesis in *C. elegans*, is negatively regulated by TRA-1 in *B. okinawaensis* as well. These results support our inference that the downstream components of sex determination are conserved among clade IV and V nematodes. Based on the RNA-seq data, TRA-1 activity could be controlled by post-translational degradation in *B. okinawaensis*, as *in C. elegans*^52^. Second, *Bok-tra-1a* is not the sole terminal regulator in the sex determination. Most strikingly, our RNA-seq data showed many structural proteins differentially expressed between wild-type males and pseudomales.

Given that this is the first intensive genetic and genomic study on the sex determination mechanisms in a nematode group greatly diverged from *Caenorhabditis* and *Pristionchus*, it provides us the critical information to understand the evolution of the sex determination pathway among nematodes, an area of much speculation. One possibility is that the pathway is derived from co-optation of a hedgehog signaling pathway^18^. In *C. elegans*, the zinc-finger transcription factor, TRA-1, is the terminal regulator of somatic sex determination and an ortholog of a Ci/Cli-like transcription factor that is the terminal regulator of the hedgehog (hh) pathway. Also, the TRA-2 transmembrane receptor that directly regulates TRA-1 directly is topologically similar to hh-receptor protein PATCHED. Since the orthologs of *C. elegans* sex determination genes *her-1* and *tra-1* have been isolated in both *Brugia malayi* and *P. pacificus*, the sex determination pathway from *her-1* to *tra-1* seemed to be co-opted in one step. However, the orthologs of *fem-1*, *tra-2*, and *her-1* are missing in the genome of *Bursaphelenchus*. If an ancestral nematode acquired the entire pathways from *her-1* to *tra-1* at once, it is unlikely that only partial genes exist in other nematode species. The other possibility proposed is that the sex determination pathway of nematodes has been evolved backwards, from the most downstream regulator (*tra-1*) to the most upstream trigger^53^ as proposed for biochemical pathways by Horowitz^54^. The findings in this study are consistent with this hypothesis. The OrthoFinder analysis showed that only a few of downstream genes (*tra-1, fem-2*, and *tra-3*) are present in *Bursaphelenchus* genome. Among these, the functional conservation of *tra-1* is confirmed, and the RNA-seq analysis showed that the gene expression of *fem-2*, *tra-3* and *mab-3* are sexually biased suggesting their participation in the sex differentiation. These genes are probably ancient in the evolution of nematode sex determination pathway. On the other hand, no upstream genes involved in X-chromosome dosage counting elements in the sex determination pathway is present in *Bursaphelenchus*, suggesting that XX/XO sex determination system and the dosage compensation mechanisms in *Caenorhabditis* have been acquired relatively recently during the process of nematode evolution. Since Mermithidae and some animal-/plant-parasitic nematode species belonging to clade I and clade IV, respectively, considered to have an ESD without a sex chromosome, there is possibility that the XX/XO system may not be the basal sex determination system among nematodes. In fact, the conservation of *C. elegans* sex determination gene orthologs in Clade IV nematodes shows that all clade IV nematode species examined do not have upstream genes such as *sdc-1*, *sdc,2*, or *sdc-3* as one-to-one orthologs (Fig. 3). Among these, *Strongyloides* spp. and *Panagrellus redivivus* has two sexes^55–57^, XO males and XX females, suggesting that XX/XO system might be evolved many times independently among nematodes. The sex determination of nematodes has been extensively studied in clade V including *Caenorhabditis* nematodes, whereas studies in other clades is very limited. Identification of upstream genes of the sex determination pathway in *B. okinawaensis* will help to understand the evolution of sex determination mechanisms among nematodes.

Genome comparison between males and females/hermaphrodites in the two *Bursaphelenchus* species revealed that structural differences in chromosomes do not contribute to sex determination. We also established the genetics of *B. okinawaensis*, enabling us to identify the first sex determination gene in Clade IV nematodes. This study using *B. xylophilus* and *B. okinawaensis* indicated the sex determination pathway of nematodes has been arisen “bottom-up” during the nematode evolution. Although we concluded that the sex differentiation in *B. okinawaensis* could be triggered by stochastic and epigenetic regulation based on many experimental evidence, the upstream genes of the sex determination pathway involved in the trigger regulation remain unclear. If we develop tools for reverse genetics for *B. okinawaensis* in the future, we will be able to clarify the sex determination in more detail. *B. okinawaensis*, which is phylogenetically distant and whose life history differs greatly from the established genetic models such as *Caenorhabditis* nematodes and *P. pacificus*, may be a powerful experimental system for studies on the evolution of development.

## Methods

### Nematode strains and maintenance

We used two strains of *B. okinawaensis* (SH1 and SH3). The SH1 strain was isolated in 2012 from an adult *Monochamus maruokai* beetle caught on Ishigaki island, Okinawa, Japan^24^. The SH3 strain was isolated in 2014 from an adult *M. maruokai* beetle caught on Iriomote island, Okinawa, Japan.

We used two strains of *B. xylophilus* (Ka4C1 and S10-P9) in this study. The Ka4C1 strain is an inbred line established by sister-brother mating over 8 generations from the Ka4 strain, originally isolated from Ibaraki, Japan, and was the strain used for *B. xylophilus* genome sequencing^27^. The inbred line S10-P9 was established from the S10 strain, which was originally isolated from Shimane, Japan^58^.

In all experiments except for the egg collecting procedure, the nematodes *B. okinawaensis* and *B. xylophilus* were propagated on the budding yeast *Saccharomyces cerevisiae*, strain W303-1A, on 1/10 malt extract agar medium (MEA) (Difco) with 4% agar containing 100 µg/mL chloramphenicol, in a 60 mm Petri dish (hereafter called “1/10 MEA-chloramphenicol plate”). For the egg collection procedure, the nematodes were propagated on the filamentous fungus *Botrytis cinerea* on MEA plates with 4% agar containing 100 µg/ml chloramphenicol, in a 90 mm Petri dish.

### Staining the germ cell with DAPI

Adult hermaphrodites and adult males propagated on 1/10 MEA-chloramphenicol plates were picked up and transferred into a droplet of 0.1 M NaCl in the well of an eight-well glass slide^25^. The worms were cut near the anterior region with a razor blade to release the gonad. The 0.1 M NaCl was completely exchanged for −20 °C methanol by pipetting, the nematode was then incubated for 5 min, and then stained with 2 µg/mL of DAPI in phosphate-buffered saline (PBS) for 10 min. After washing twice with PBS, the worms were mounted with Vectashield onto glass slides (Vector Laboratories Inc., USA). DAPI-stained images were obtained with a confocal laser-scanning microscope (LSM5 exciter, Carl Zeiss).

### Environmental effect on the sex ratio of *B. okinawaensis*

To investigate the impact of environment and developmental history on the sex determination of *B. okinawaensis*, the sex ratio after treatment of five different stimuli (temperature, culture period, heat shock, ethanol shock, and population density), and two different developmental states (dauer and starvation) were examined.

#### Temperature

A single mid- to late-L4 hermaphrodite was picked from a synchronized plate which was prepared according to Shinya et al^24^. and transferred to a 1/10 MEA-chloramphenicol plate with yeast lawn. The plate was incubated for 7 days and 14 days at 20, 25, or 30 °C, and then the number of hermaphrodites and male progeny were counted. This experiment was performed in ten biological replicates for each.

#### Heat shock

Five mid- to late-L4 hermaphrodites were picked from a synchronized plate and transferred to a 1/10 MEA-chloramphenicol plate with yeast lawn. Plates were exposed at 40 °C for 2 or 4 h and then returned to 25 °C. The plates were incubated for 4–5 days at 25 °C, and then the number of hermaphrodite and male progeny were counted. This experiment was performed in ten biological replicates.

#### Ethanol shock

Mid- to late-L4 hermaphrodites were prepared by the above-mentioned synchronization method. The worms were picked and transferred to 10% ethanol in ddH_2_O. After 1 h ethanol treatment at 25 °C, the worms were washed with ddH_2_O three times, and five worms were returned to each 1/10 MEA-chloramphenicol plate with yeast lawn. The plate was incubated for 4–5 days at 25 °C, and then the number of hermaphrodite and male progeny were counted. This experiment was performed in ten biological replicates.

#### Population density

50 mid- to late-L4 hermaphrodites were picked from a synchronization plate and transferred to a nutrient-rich agar plate (1/1 MEA-chloramphenicol with 0.5% peptone). The plate was incubated at 25 °C for 36 h to allow the hermaphrodites to lay eggs, and then the adult hermaphrodites were removed from plate. After 3 days incubation at 25 °C, the number of F1 hermaphrodites and males was counted. This experiment was performed in ten biological replicates.

#### L4 larval starvation

Mid- to late-L4 hermaphrodites were prepared by the above-mentioned synchronization method. The worms were then transferred to an unseeded water agar plate and incubated at 25 °C. After 20 h incubation with the absence of food, a single worm was transferred to a 1/10 MEA-chloramphenicol plate with yeast lawn. The plate was incubated for 4–5 days at 25 °C, and then the number of hermaphrodite and male progeny were counted. This experiment was performed in 25 biological replicates.

#### Dauer recovery

Dauer larvae were collected from 2-week-old worm cultures into sterilized ddH_2_O in a glass Petri dish. The dauer larvae were incubated at 25 °C for two weeks under starvation conditions to make worms easier to recover from the dauer stage. Five dauer larvae were picked and transferred to a 1/10 MEA-chloramphenicol plate with yeast lawn. The plate was incubated for 5–6 days at 25 °C, and then the number of hermaphrodites and male progeny (F1) were counted. This experiment was performed in 35 biological replicates. Then, five F1 hermaphrodites were picked and transferred to each 1/10 MEA-chloramphenicol plate with yeast lawn. The plate was incubated for 5–6 days at 25 °C, and then the number of hermaphrodite and male progeny (F2) were counted. This experiment was performed in 10 biological replicates.

### Detection of endosymbiont microbes in *B. okinawaensis*

To investigate whether *Bursaphelenchus* nematodes have symbiotic microbes that are able to manipulate their host sex-determination system (such as *Wolbachia)*, we examined if the sex ratio can be changed by treatment of antibiotics. Antibiotics tested were tetracycline, chloramphenicol, and kanamycin. The L4 hermaphrodites of *B. okinawaensis* SH1 were collected and soaked in each antibiotic solution (20 µg/ml tetracycline, 100 µg/mL chloramphenicol, or 50 µg/mL kanamycin) for 2 h under darkness at room temperature. Then, five worms were picked and transferred to a 1/10 MEA plate containing 20 µg/ml tetracycline, 100 µg/mL chloramphenicol, or 50 µg/mL kanamycin with yeast lawn. The plate was incubated for 5–6 days at 25 °C under darkness, and then the number of hermaphrodite and male progeny were counted. This experiment was performed in 10 biological replicates for each treatment.

Adult SH1 *B. okinawaensis* hermaphrodites were screened for the presence of endosymbiotic bacteria. DNA of *B. okinawaensis* was extracted and purified using a Wizard Genomic DNA Purification kit (Promega) according to the manufacturer’s protocol, with a small modification in the worm lysis step. Adult hermaphrodites of *B. okinawaensis* were washed off from the *Botrytis cinerea* fungal culture plates and washed 3 times by spinning down in sterilized ddH_2_O to get rid of fungal contamination. Then 50 µl of worm pellet was transferred into 600 µl of chilled Nucleic lysis solution contained in the kit, supplemented with 20 µl of 20 mg/ml proteinase K, and incubated for 4 h at 55 °C with continuous shaking on a shaker. As a positive control, DNA was extracted from *E. coli* OP50 using the same kit. Detection was based on the amplification of 16 S rRNA gene fragments using bacterial universal primers. Two sets of universal primers, 8 F/1391 R and 27 F/1492 R, were used in this experiment (Table S5). PCR amplifications were performed in a final volume of 50 µl, containing 5 µl of DNA, 5 µl of 10x reaction buffer, 4 µl of 2.5 mM dNTP mix, 31.75 µl of ddH_2_O, 0.25 µl of ExTaq polymerase (Takara), 2 µl of 10 µM forward primer, and 2 µl of 10 µM reverse primer. Amplification was performed in an S1000 thermal cycler (Bio-Rad), using the following cycling conditions: 98 °C for 30 sec, followed by 30 cycles of 10 sec at 98 °C, 30 sec at 48 °C, 1 min at 72 °C. PCR products were then analyzed using gel electrophoresis on a 1.5% agarose gel.

### Genome sequencing of *B. okinawaensis* SH1 strain and *B. xylophilus* Ka4C1 strain

#### Isolation of DNA and RNA

To extract genomic DNA and RNA from *B. okinawaensis*, SH1 strain and *B. xylophilus* Ka4C1 strain, nematodes were propagated on the filamentous fungus *Botrytis cinerea* on MEA plates with 4% agar containing 100 µg/ml chloramphenicol in a 90 mm Petri dish. After one week of incubation at 25 °C, nematodes were collected and washed five times with sterilized ddH_2_O. For DNA extraction, a Wizard Genomic DNA Purification Kit (Promega, Madison, WI) was used, following the manufacturer’s protocol. The genomic DNA was then treated with RNase A to remove any RNA fragments present in the sample. For total RNA extraction, nematodes were frozen in liquid nitrogen and then ground in a mortar and pestle to make powder, then total RNA was extracted and purified using an RNeasy Plus Mini Kit (Qiagen) according to the manufacturer’s protocol.

#### Sequencing and assembly

Paired-end sequencing libraries were prepared using the Nextera DNA Sample Prep kit (Illumina) according to the manufacturer’s instructions. Mate-paired libraries (3 kb) were constructed using the Nextera Mate-Paired Library Construction kit (Illumina). Libraries were sequenced on the Illumina HiSeq 2500 sequencer using the Illumina TruSeq PE Cluster kit v3 and TruSeq SBS kit v3 (151 cycles × 2). The raw sequence data were analyzed using the RTA 1.12.4.2 analysis pipeline and were used for genome assembly after the removal of adapter, low quality, and duplicate reads.

Ten μg of *B. xylophilus* genomic DNA was used to prepare PacBio ~20 kb insert size library. A total of 10 SMRT cells were sequenced on a PacBio RSII using P6-C4 chemistry generating 6.9 Gb of data representing 92× genome depth of coverage.

For RNA-seq analyses, one hundred ng of total RNA was used to construct an Illumina sequencing library using the TruSeq RNA-seq Sample Prep kit according to the manufacturer’s recommended protocols (Illumina). The libraries were sequenced for 101 or 151 bp paired-ends on an Illumina HiSeq 2500 sequencer using the standard protocol (Illumina).

A k-mer count analysis was done using K-mer Counter (KMC ver.3.1.0)^59^, on the paired-end Illumina data. Only the first read was used to avoid counting overlapping k-mers. Ploidy estimations were performed using Smudgeplot (v0.2.5)^60^.

*B. okinawaensis* assembly (v1.0) was generated using the Illumina short reads. The paired-end and mate-pair reads were assembled using the MaSuRCa assembler v2.2.1^61^ with options (CA_PARAMETERS: ovlMerSize = 30, cgwErrorRate = 0.15, utgErrorRate = 0.015, ovlMemory = 4GB). Gapfiller (v2.1.2)^62^ and Image (v2.4.1)^63^ were run on the assembly to fill gaps. Base correction was then performed with the Illumina pair-end reads using ICORN2 (version 2)^64^ with 3 iterations.

*B. xylophilus* initial assembly (v3.0) was generated from PacBio reads were produced using Canu (v1.3). The assembly was improved using the Quiver module in the SMRT Analysis pipeline (version 2.3.0; http://www.pacbiodevnet.com/SMRT-Analysis/Software/SMRT-Pipe) and base correction was performed with the Illumina pair-end reads using ICORN2 (version 2)^64^ with three iterations.

To generate chromosome-scale assemblies of *B. xylophilus* (v5.0)^29^ and *B. okinawaensis* (v2.0) ^28^, Oxford Nanopore long reads were assembled using Flye (v2.8.3-b1695)^65^ and was further scaffolded using 3D-DNA pipeline (v201008)^66^ with HiC sequencing data. The sequence data and genome assemblies are available under the ENA Project Nos. PRJEB40022 and PRJEB40023 for *B. xylophilus* and *B. okinawaensis*, respectively.

To predict protein-coding genes, Augustus (v. 3.0.1) was trained for *B. okinawaensis* based on a training set of ~1000 non-overlapping, non-homologous, and manually curated genes. For *B. xylophilus*, previously trained Augustus parameters^67^ were used to predict gene models on the new assembly. RNA-seq reads mapped to the genomes using Hisat2 were used to generate the intron hints and the exonpart hints. The trained versions of Augustus were run using all the hints for that species as input to predict 17,283 and 14,844 protein-coding genes for *B. xylophilus* and *B. okinawaensis*, respectively. To establish orthology relationships among *B. xylophilus*, *B. okinawaensis,* and *C. elegans*, non-redundant proteomes (protein sets containing only longest isoforms) were grouped into orthologue groups using OrthoFinder (v0.2.8)^68^ with default options.

Sex-specific Illumina sequencing libraries were generated from 800–900 nematodes from each sex stage of *B. okinawaensis* and *B. xylophilus* using the NuGen Ovation Ultralow Library System V2 library preparation kit. The libraries were sequenced for 151 bp paired-ends on an Illumina HiSeq 2500 sequencer using the standard protocol (Illumina). The short reads were mapped to the reference genome (v5.1 for *B. xylophilus* or v2.0 for *B. okinawaensis*) using SMALT v0.7.4 (https://www.sanger.ac.uk/resources/software/smalt/) with options –*x* (each mate is mapped independently) and –*y* 0.8 (mapping to the region of highest similarity in the reference genome at a similarity threshold >80%). The depth of mapped read coverage at each genome position was calculated using the Mosdepth v0.2.9^69^ and summarized by 5-kb window. Copy number analysis was conducted using CNVkit v0.9.9^70^ and copy number variations were called under parameters (-m threshold −*t* = −2,−0.4,0.3,0.8 --filter ci).

SNPs were called for each sex stage using the Genome Analysis Toolkit (ver. 3.3.0)^71^ by following the GATK Best Practice for germline short variant discovery. SNP density was calculated using the vcftools v0.1.13^72^.

A k-mer count analysis was done using K-mer Counter (KMC ver.3.1.0)^59^, on the paired-end Illumina data. Male-specific contigs were detected based on k-mer by DiscoverY (v2019)^31^ with options (--mode female+male --female_kmers_set --kmer_size 31”).

### Detection of sex determination locus using SNP primers in *B. xylophilus*

#### SNP discovery

Illumina short reads of two *B. xylophilus* strains (the reference strain Ka4C1 and S10-P9) were aligned to the reference genome (v3.0) using SMALT v0.7.4 as described above. SNPs were called using the Genome Analysis Toolkit (ver. 3.3.0)^71^ by following the GATK Best Practice for germline short variant discovery.

#### SNP primer design

To map the sex determination locus in *B. xylophilus*, SNPs were analyzed using PCR-based cleaved amplified polymorphic sequence (CAPS) assay. Primer sets for the CAPS assay were designed for each linkage group using Galaxy python scripts find_CAPS.py (v2012) and design_primers.v3.py^73^. Each primer set was designed for a different chromosome or region (Supplementary Table 4).

#### Detection of sex determination locus

To investigate if the way the mechanism works is that there is a dominant male determining locus or recessive female determining locus, an unmated Ka4C1 adult female was mated with a S10-P9 male, and F1 progeny was obtained. Then, an F1 hybrid male was backcrossed with a new Ka4C1 female and F2 progeny was obtained. To investigate the presence of a dominant female determining locus or recessive male determining locus, an unmated Ka4C1 female was mated with an S10-P9 male, and F1 progeny was obtained. Then, an F1 hybrid female was backcrossed with a Ka4C1 male and F2 progeny was obtained. In both cases, 30 F2 females and 30 F2 males were picked and pooled into separate tubes, each containing 60 μL DirectPCR lysis reagent (Viagen) with 1 mg/ml proteinase K. The worms were lysed by incubation at 60 °C for 20 min and then the proteinase was inactivated by incubation at 95 °C for 10 min. 1 μL of each DNA template was then added to a 24 μL of PCR mix containing 15 μL of ddH_2_O, 5 μL of 5× PrimeSTAR GXL Buffer, 2 μL of dNTP (2.5 mM each), 0.75 μL of primer (10 μM each), and 0.5 μL of PrimeSTAR GXL DNA polymerase. PCR reactions were performed using the following cycling conditions: 35 cycles of 10 sec 98 °C, 15 sec of 56 °C, 68 °C 80 sec. After amplification, PCR products were digested with the restriction enzyme BamHI-HF in a final volume of 40 μL (25 μL of PCR product, 10.7 μL of ddH2O, 4.0 μL of CutSmart buffer, 0.3 μL of BamHI-HF) at 37 °C for 6–8 h. Samples were then loaded onto a 2.0 % agarose gel. If there is a sex determination locus on a chromosome, we should find a difference in the DNA band pattern between F2 males and females with a specific primer set.

### F2 mutagenesis screens

Mutations were generated by treatment with EMS as described by Shinya et al.^24^, with some modifications. Young adult hermaphrodites of the SH1 strain were collected and washed three times with sterilized ddH_2_O. The nematodes were suspended and incubated in 30–50 µM EMS in M9 buffer for 4 h at room temperature. Nematodes were then washed three times with M9 buffer and transferred to the edge of a 1/10 MEA-chloramphenicol plate with yeast lawn to produce F1 self-progeny. After three days of incubation at 25 °C, four L3-L4 hermaphrodites from the F1 generation were transferred onto a new yeast plate and allowed to lay eggs. Each plate was subsequently scored for the percentage of F2 males. When at least two males were observed per plate in the F2 screening, 20–30 sister hermaphrodites were cloned and scored for the presence of F3 males.

### Chromosome mapping for candidate genes using SNP markers

#### SH3 strain isolation

The SH3 strain of *B. okinawaensis* was isolated in July in 2014 from adult female of the beetle *Monochamus maruokai* from the Iriomote island, Okinawa, Japan. This is the first strain of *B. okinawaensis* isolated from outside the Ishigaki island. The Iriomote islands are located about 20 km southwest of the Ishigaki island, and about 450 km southwest of the main Okinawa Islands.

#### Whole-genome resequencing of SH3 strain for the genome-wide SNP discovery

To extract genomic DNA from the SH3 strain, nematodes were propagated on the filamentous fungus *B. cinerea* on MEA plates with 4% agar containing 100 µg/ml chloramphenicol in a 90 mm Petri dish. After one week of incubation at 25 °C, nematodes were collected and washed five times with sterilized ddH_2_O. For DNA extraction, a Wizard Genomic DNA Purification Kit (Promega) was used, following the manufacturer’s protocol. The genomic DNA was then treated with RNase A to remove any RNA fragments present in the sample. Illumina short reads were obtained for *B. okinawaensis* SH3 strain and SNPs were called as described above.

#### SNP mapping

Sperm-depleted old SH1 hermaphrodites with a heterozygous Tra mutation which was isolated in this study were crossed with SH3 males. 30 Tra (homozygous) males and 30 wild-type (WT) hermaphrodites from among the self-progeny of *Bok-tra(sy867* or *sy868)*/SH3 heterozygote hermaphrodites were picked and pooled into separate tubes, each containing 60 μL DirectPCR lysis reagent (Viagen) with 1 mg/ml proteinase K. The worms were lysed by incubation at 60 °C for 20 min and proteinase was subsequently inactivated by incubation at 95 °C for 10 min. 1 μL of each DNA template was then added to 24 μL of PCR mix containing 15 μL of ddH2O, 5 μL of 5× PrimeSTAR GXL Buffer, 2 μL of dNTP (2.5 mM each), 0.75 μL of a 10 μM solution for each primer, and 0.5 μL of PrimeSTAR GXL DNA polymerase. PCR reactions were performed using the cycling conditions: 35 cycles of 10 sec of 98 °C, 15 sec of 56 °C, 68 °C of 80 sec. After amplification, PCR products were digested with the restriction enzyme BamHI-HF in a final volume of 40 μL (25 μL of PCR product, 10.7 μL of ddH_2_O, 4.0 μL of CutSmart buffer, 0.3 μL of BamHI-HF) at 37 °C for 6–8 h. Samples were then loaded onto a 2.0 % agarose gel. Each mutant sample was loaded next to its non-mutant control. To assign mutations to chromosome regions, two representative SNP markers per chromosome region were tested (Supplementary Table 7). To assign linkage of a mutation to three regions (reg0008 on chr2, 0023 on chr3, and 0111 on chr5), two (for reg0008 and 0023) or three (for reg0023) representative SNP markers were generated (Supplementary Table 8), and use to examine the linkage with the *Bok-tra(sy867)* mutant. In this case, PCR products were digested with the restriction enzyme DraI HF in a final volume of 40 μL (25 μL of PCR product, 10.7 μL of ddH_2_O, 4.0 μL of CutSmart buffer, 0.3 μL of DraI) at 37 °C for 8 h followed by 80 °C for 20 min.

#### Complementation test

Because Tra (*tra*/*tra*) males are infertile, heterozygous *Bok-tra (sy867)* mutant hermaphrodites were used for the complementation test. First, the sperm-depleted old hermaphrodite heterozygous mutants were crossed with SH1 WT males. F1 males which were phenotypically wild-type were picked and used for subsequent cross-mating with the heterozygous *Bok-tra (sy868)* mutant hermaphrodites. If pseudomales were generated in F2, the mutation of *Bok-tra (sy867)* and *Bok-tra (sy868)* should be generated in the same gene.

#### Mutation identification by whole-genome sequencing

10 of the adult pseudomales of homozygous Tra (*tra*/*tra*) mutants and 10 of the hermaphrodites (*+/+*) were picked and pooled into separate tubes, each containing 5 μL DirectPCR lysis reagent (Viagen) with 1 mg/ml proteinase K. The worms were lysed by incubation 60 °C for 30 min. Then, the extracted genome was amplified using the illustra Ready-To-Go GenomiPhi V3 DNA amplification kit. The 3 μL of worm lysis solution was transferred into a new 0.2 ml tube and 7 μL of ddH_2_O and 10 μL of 2× denaturation buffer were added. Denaturation was performed by heating at 95 °C for 3 min and then cooled on ice for 5 min. 20 μL of denatured template DNA was added to each cake containing all components for amplification and incubated at 30 °C for 2 h followed by inactivation at 65 °C for 10 min then cooling on ice. The amplified genome was purified using QiaAmp DNA mini kit according to the manufacturer’s protocol. The final elution was performed with ddH_2_O.

### RNA-seq analysis for *Bok-tra-1(sy867)* mutant

Total RNA was prepared separately from one-day-old adult pseudomales (*tra/tra*) and one-day-old adult hermaphrodites (*tra/+* or + */+*) in *Bok-tra-1(sy867)*. 250 of each type of nematode were handpicked from yeast plates for each of three biological replicates and washed five times with sterilized ddH_2_O in 1.5 mL Eppendorf tubes. The worm suspension in the tube was frozen in liquid nitrogen 350 μl of buffer RLT in RNeasy Mini Kit (Qiagen) was added and then the tube was frozen again. The worms were ground in the frozen tube with a pellet pestle and total RNA was extracted and purified using an RNeasy Plus Mini Kit (Qiagen) according to the manufacturer’s protocol. RNA-seq libraries were prepared using NEB Next Ultra RNA library prep kit for Illumina (New England Biolabs, E7530). RNA-seq libraries were sequenced on Illumina Hiseq 2500 in single-end mode with a read length of 100 nt. RNA-seq reads were mapped to the reference genomes using Hisat2^74^ v2.1.0 with the default parameters. The mapped read count of each gene was calculated using HTSeq^75^ v0.9.1 with options (−s no, −a 10, −m union) and differential expression analyses were performed using EdgeR v3.2.4^76^. A transcript was identified as differentially expressed in a pairwise comparison if the following criteria were met: false discovery rate (FDR) ≤ 0.01 and fold change ≥ 2.0. FPKM values were calculated using Cufflinks packages v2.2.1^77^ and used to generate gene expression heatmaps.

### Reporting summary

Further information on research design is available in the Nature Research Reporting Summary linked to this article.

## Supplementary information

Supplementary Information

Description of Additional Supplementary Files

Supplementary Data 1

Supplementary Data 2

Supplementary Data 3

Supplementary Data 4

Supplementary Data 5

Supplementary Data 6

Supplementary Data 7

Supplementary Data 8

Reporting Summary

## Data Availability

All sequence data generated during and/or analyzed during the current study have been deposited at DDBJ/ENA/GenBank under BioProject accession PRJEB40022, PRJEB40023, and PRJDB10466 The reference genomes, v5.1 for *B. xylophilus* and v2.0 for *B. okinawaensis* are available from GenBank assembly accessions GCA_904066235.2 and GCA_904066225.2, respectively. All other relevant data are available from the authors. Supplementary Data 1–6 detail the genes differentially expressed between males and hermaphrodites, between wild-type and tra mutant males, between wild-type and tra mutant hermaphrodites, and the Gene Ontology analysis for each. Supplementary Data 7–8 list the genes in two clusters that are commonly upregulated or commonly downregulated in *tra* mutants of both *B. okinawaensis* and *C. elegans* based on their orthology relationships. Source data are provided in this paper.

## Source data

Source Data
