# Supplementary material for: Possible stochastic sex determination in Bursaphelenchus nematodes
Source: Nat Commun. 2022 May 11;13:2574. doi: 10.1038/s41467-022-30173-2 (PMC9095866; doi:10.1038/s41467-022-30173-2)
Supplement: Supplementary file 1 — Supplementary Information [file 41467_2022_30173_MOESM1_ESM.pdf]

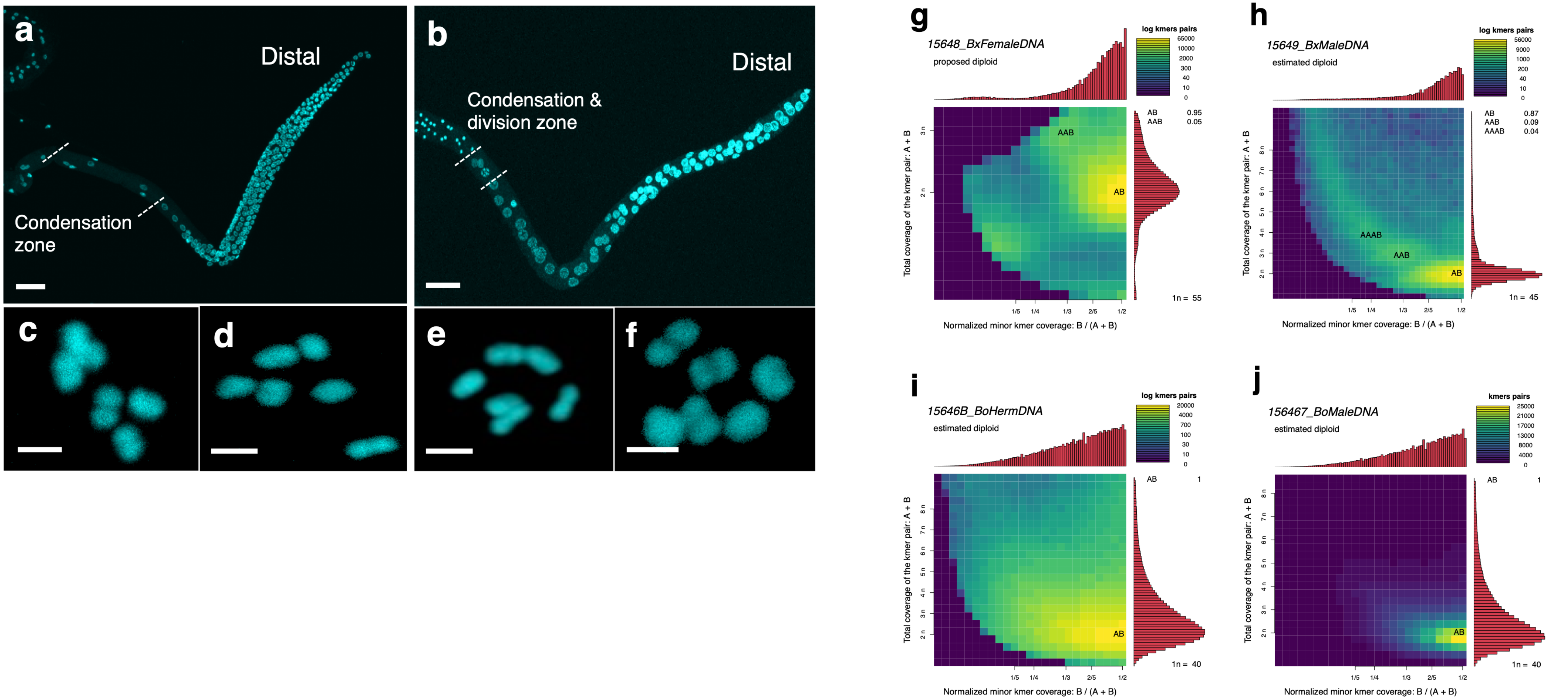

Supplementary Figure 1. Projection of a dissected hermaphrodite (a) and male (b) germ line in *B. okinawaensis* stained with DAPI. Enlarged images of DAPI-visualized bivalents in Diakinesis stage during meiosis in hermaphrodite (c), (d) and male (e), (f). Scale bars: (a) (b) = 20 µm, (c)(d)(e)(f) = 2 µm. Haplotypic K-mer analysis of the sex specific short reads using Smudgeplots for *B. xylophilus* female (g), male (h), *B. okinawaensis* hermaphrodite (i), and male (j).

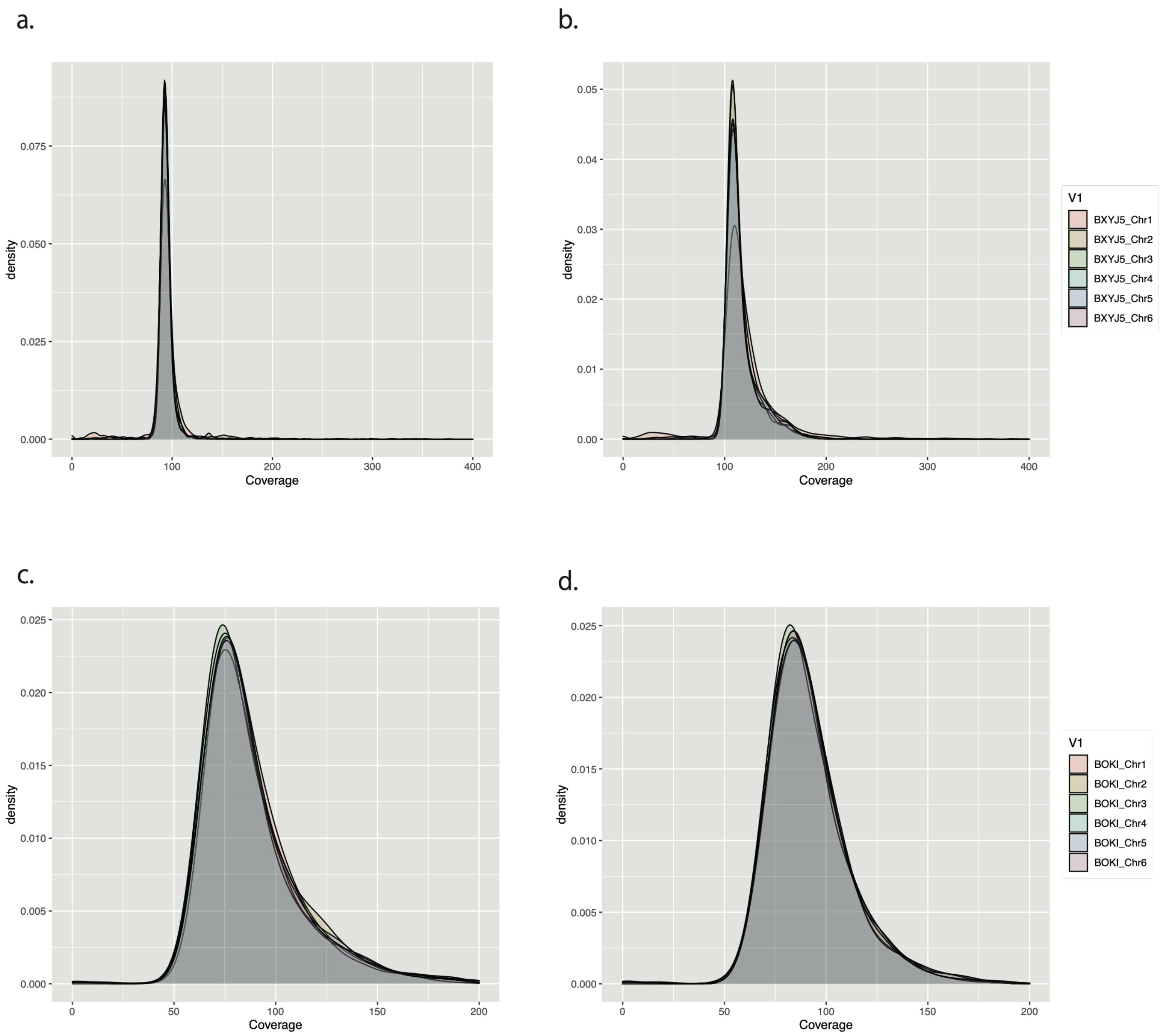

Supplementary Figure 2. Histograms of depth distribution of the sex-specific reads calculated using a 5-kb window along each chromosome for *B. xylophilus* male (a), female (b), and *B. okinawaensis* male (c) and hermaphrodite (d).

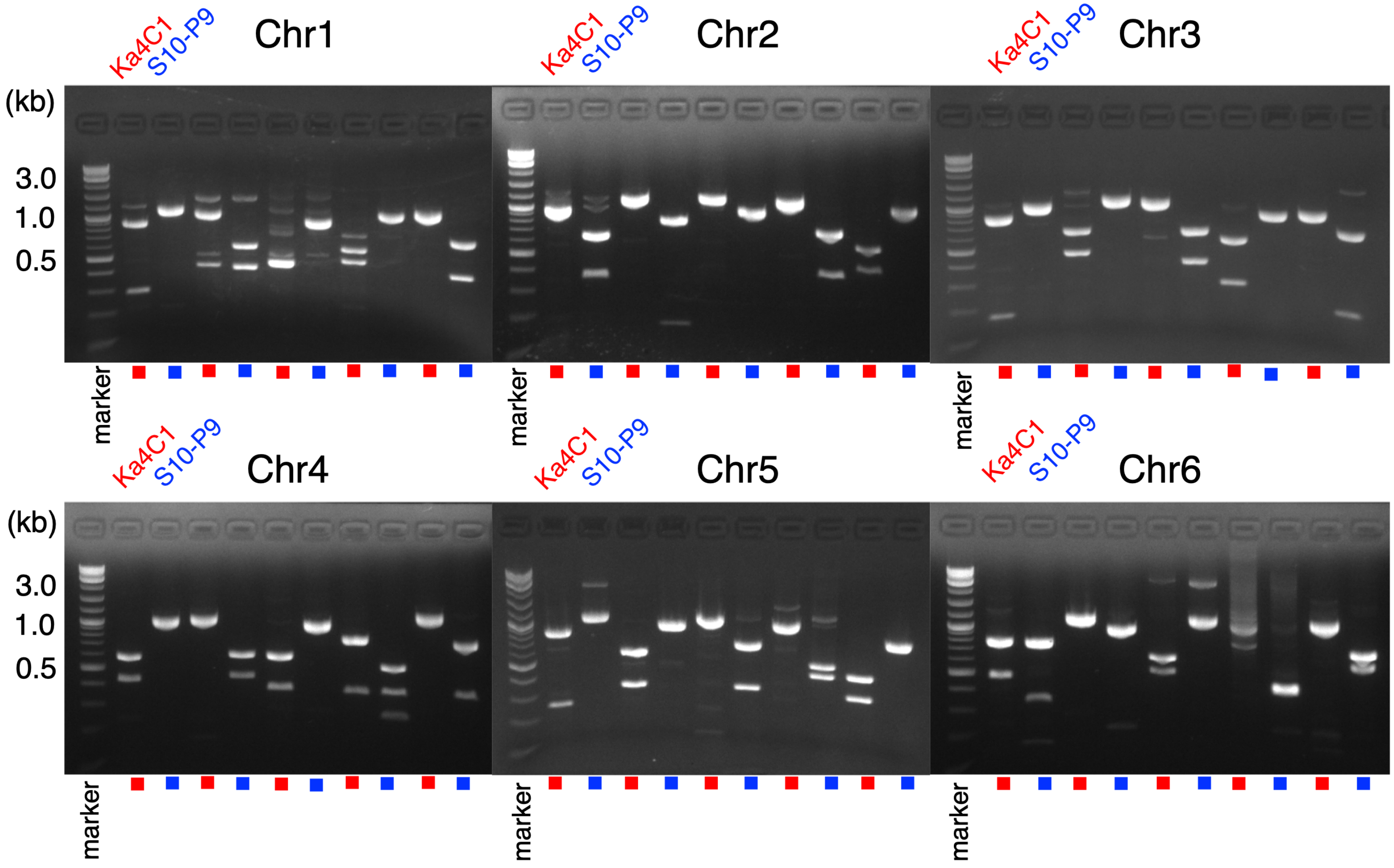

Supplementary Figure 3. Results from inbred strains of *B. xylophilus* Ka4C1 and S10-P9 genotypes after BamHI cut. 30 Ka4C1 adults including both sexes and 30 S10-P9 adults including both sexes were lysed in 60μl DirectPCR lysis reagent and served as the DNA template for the 30 PCR reactions covering all six chromosomes.

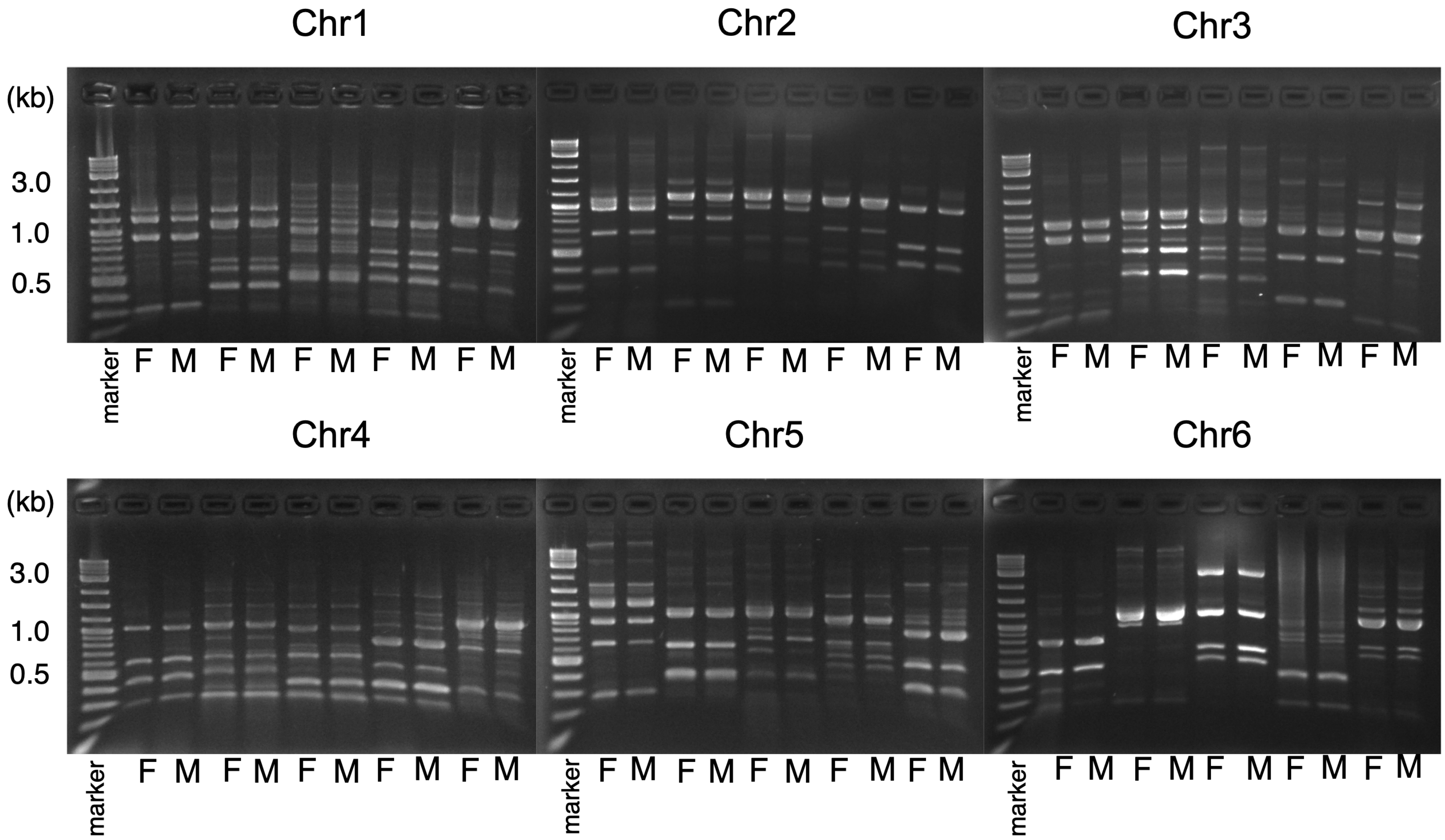

Supplementary Figure 4. SNP mapping for detecting a dominant male determining locus or recessive female determining locus in *B. xylophilus*. Each pair of lanes shows results from the SNP for each primers, using either the adult females (F) or adult males (M) DNA template after F1 male backcross with Ka4C1 female.

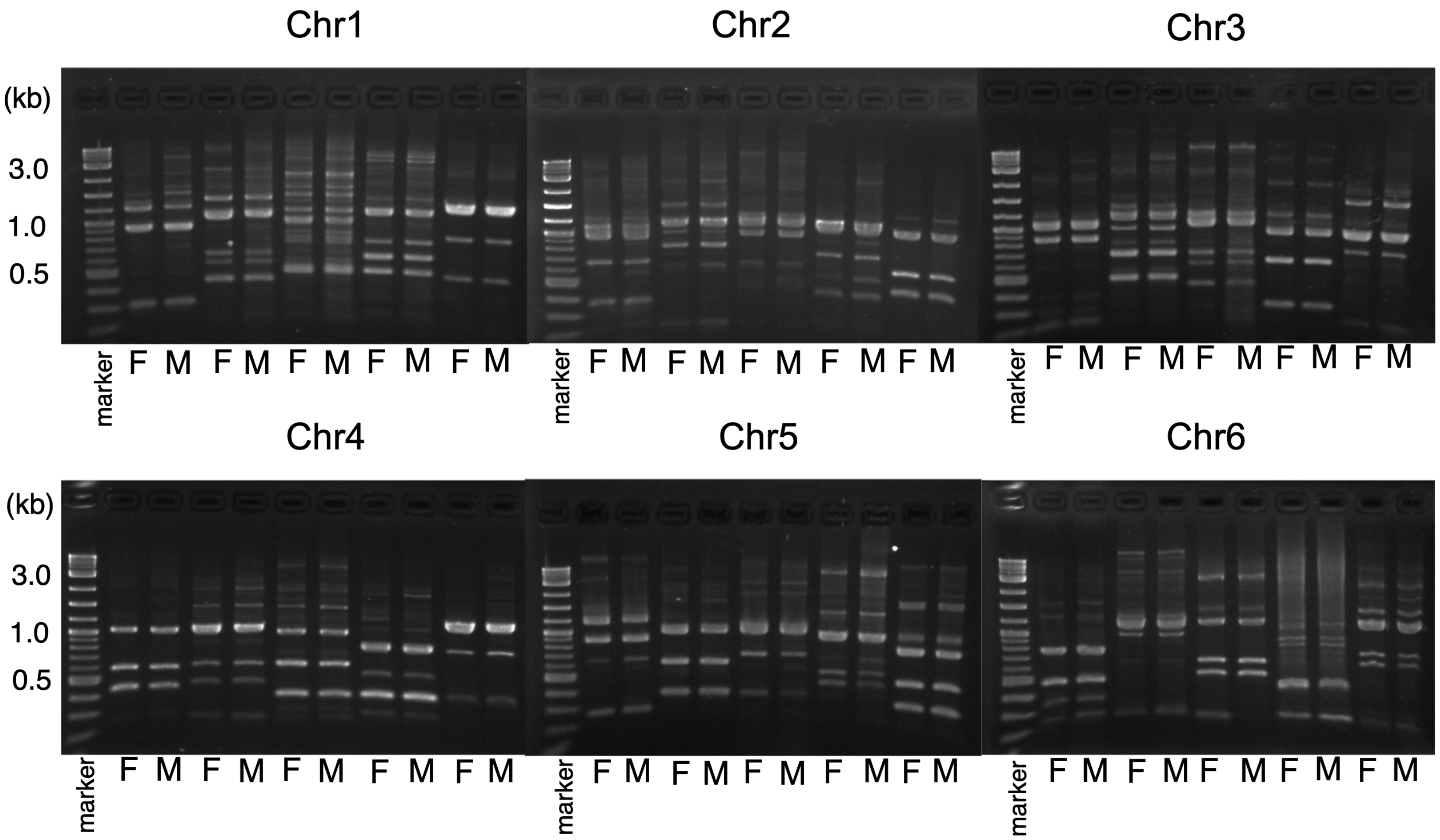

Supplementary Figure 5. SNP mapping for detecting a dominant female determining locus or recessive male determining locus in *B. xylophilus*. Each pair of lanes shows results from the SNP for each primer pair, using either the adult females (F) or adult males (M) DNA template after F1 female backcross with Ka4C1 male.

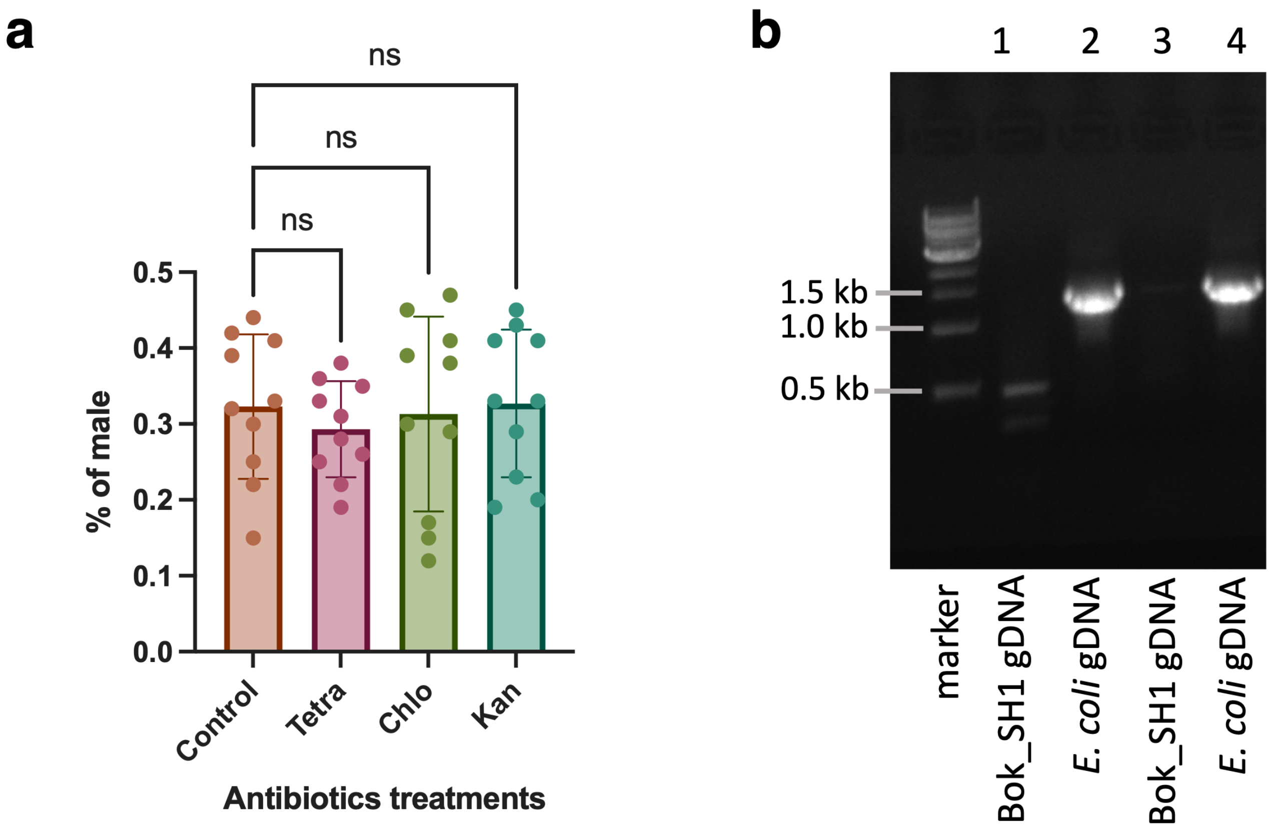

Supplementary Figure 6. Percentage of males in *B. okinawaensis* on culture media with antibiotics (a). The experiment was performed in 10 biologically independent replicates for each treatment. Data are presented as mean values ± standard deviation. ns for control versus tetracycline, chloramphenicol, and kanamycin indicates *P*=0.4175, *P*=0.8453, and *P*=0.9270, respectively (unpaired *t*-test). Confirmation of the presence of endosymbiont bacteria by PCR reaction in 1.5% agarose gel electrophoresis with bacteria universal primers, 8F and 1391R, using DNA of *B. okinawaensis* (1) and *Escherichia coli* OP50 (2) and with primers, 27F and 1492R, using DNA of *B. okinawaensis* (3) and *E. coli* OP50 (4) (b).

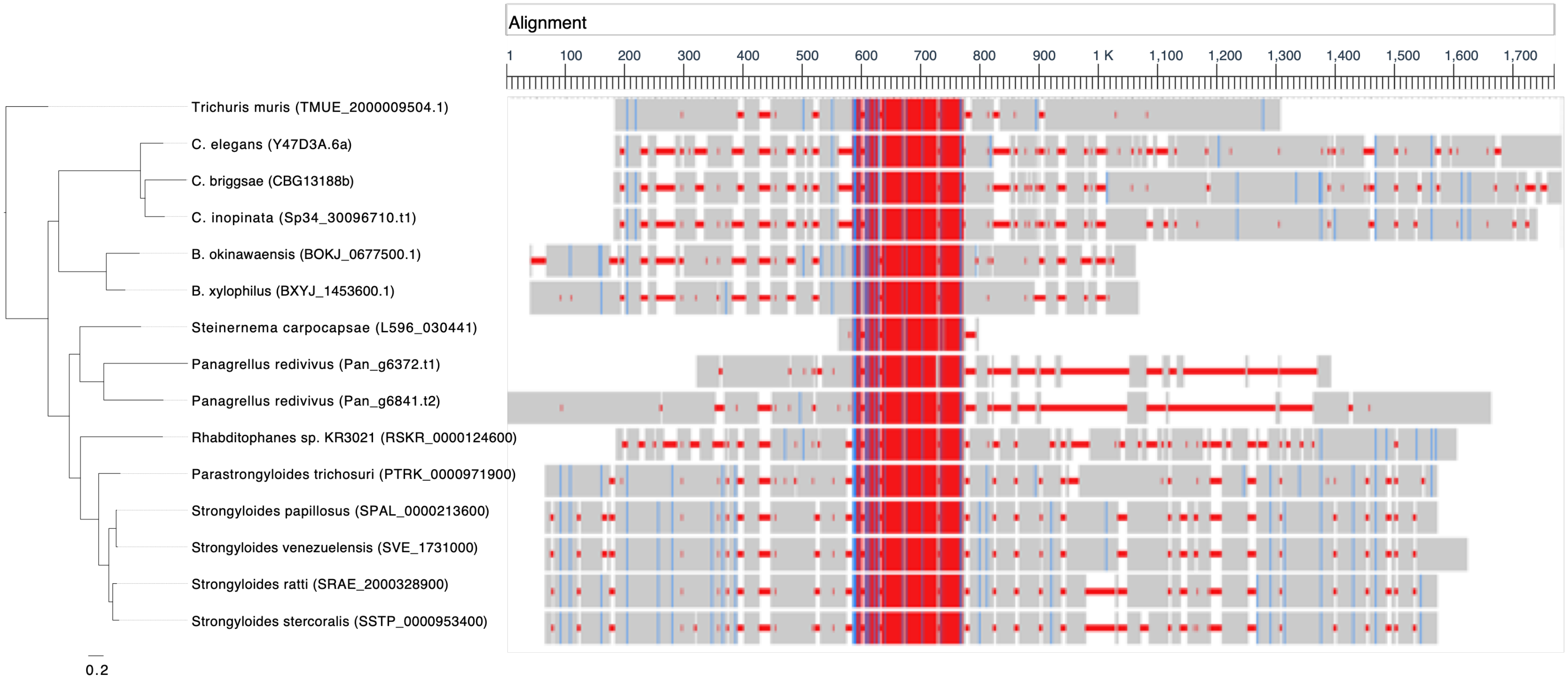

Supplementary Figure 7. Multiple alignment of amino acid sequences of TRA-1 orthologues in CladeIV with *Caenorhabditis* spp. (Clade V) and *Trichuris muris* (Clade I) as outgroups.

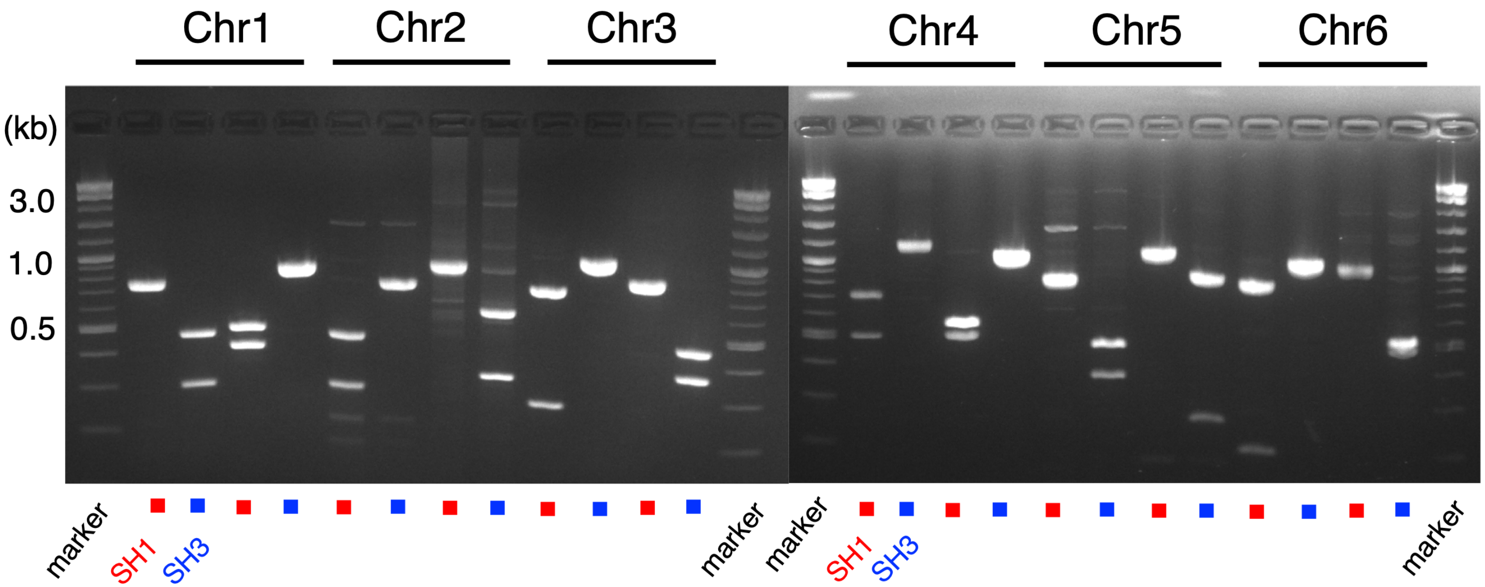

Supplementary Figure 8. Results from *B. okinawaensis* SH1 strain and SH3 strain genotypes after BamHI cut. 30 SH1 adult hermaphrodites and 30 SH3 adult hermaphrodites were lysed in 60µl DirectPCR lysis reagent, and served as the DNA template for the 30 PCR reactions covering all six chromosomes.

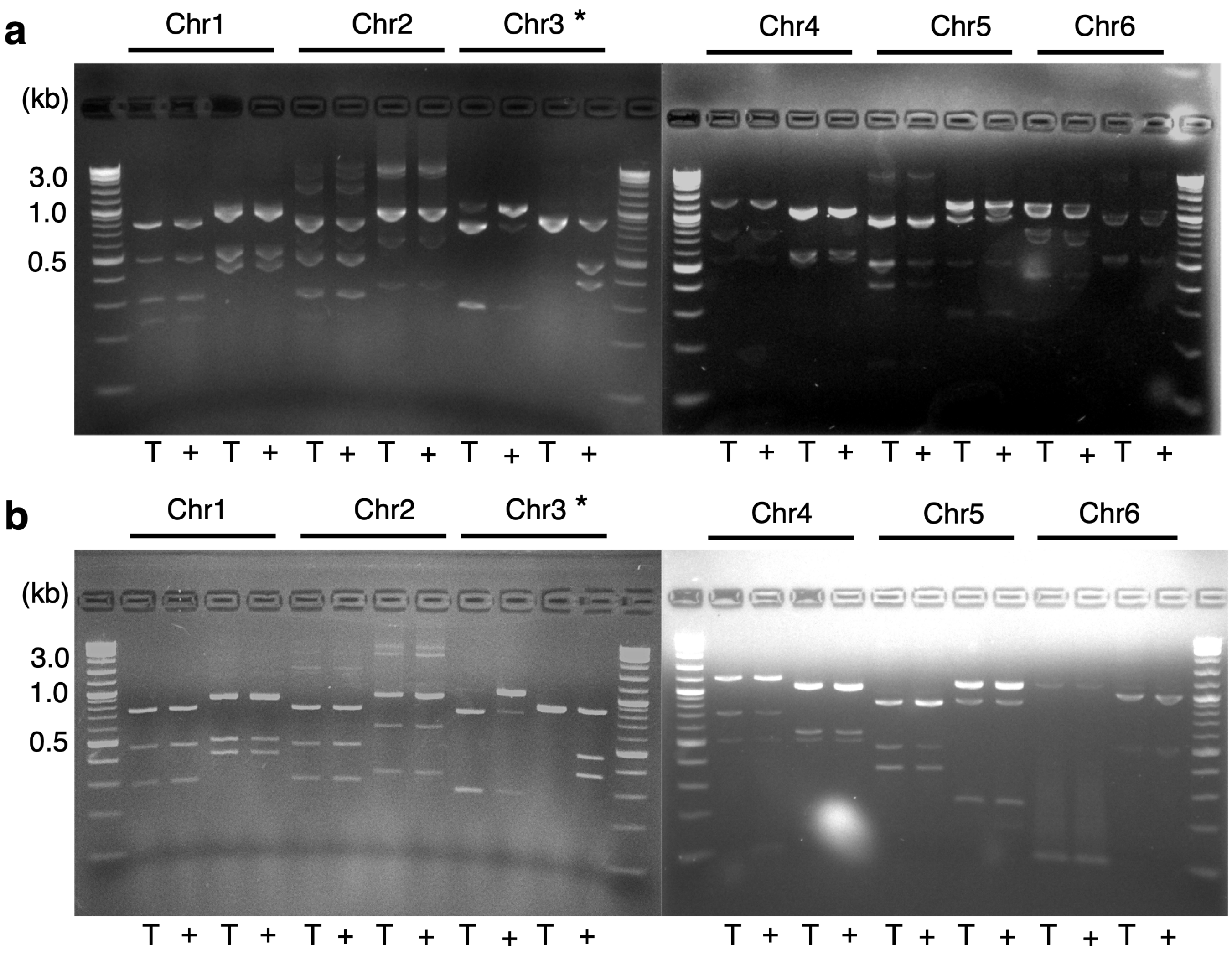

Supplementary Figure 9. Chromosome mapping for *Bok-tra(sy867)* (a) and *Bok-tra(sy868)* (b). Each pair of lanes shows results from the SNP for each primers, using either the adult pseudomale of Tra (T) or adult hermaphrodite of wild type (+) DNA template. Linkage is visible as an increase in the proportion of SH1 DNA in Tra lanes compared to the wild type lanes, and is visible on Chr3* in both primer sets.

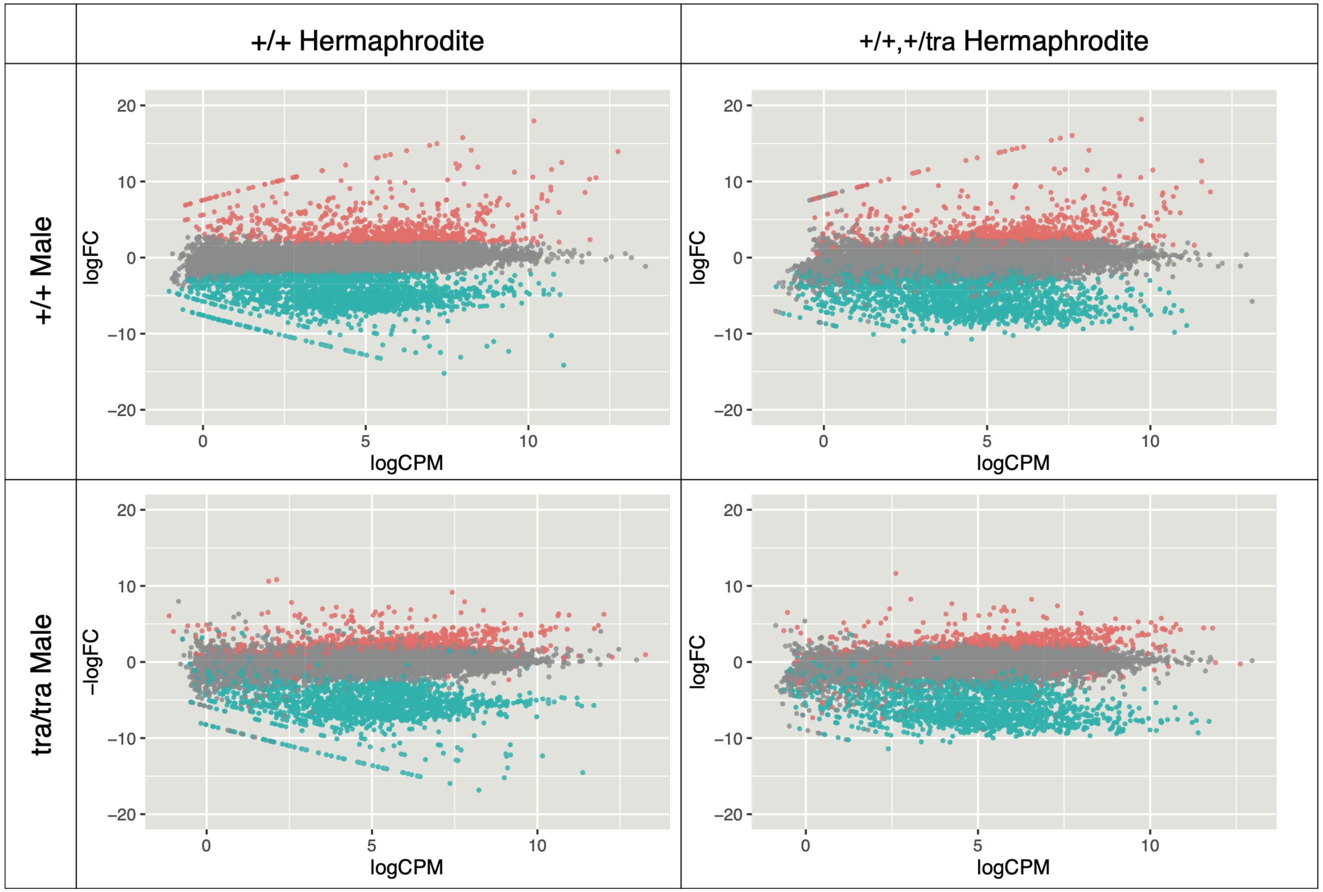

Supplementary Figure 10. MA plots showing the relationship between logCPM (the logarithm of counts per million reads) and logFC (the log-transformed fold change in gene expression) across the genes. Comparisons were performed between hermaphrodite and male using wildtype and *tra-1* mutant *B. okinawaensis*. Each gene is represented by a dot. Genes upregulated in hermaphrodites (feminine genes) and upregulated in male (masculine) in the wildtype comparison were shown in red and blue, respectively, in each MA plot.

Table S1. Chromosome-level assemblies of *B. xylophilus* and *B. okinawaensis* genomes

	B. xylophilus	*B. okinawaelnsis*
BioProject accession no.	PRJEB40022	PRJEB40023
Assembly size (Mbp)	78.3	70.0
No. of chromosomes	6	6
Unassigned scaffold number	5	1
Unassined scaffold length (kb)	50	6
G+C content (%)	40.4	36.17
Repeat contents (%)	29.27	14.27
Number of genes	17111	15500
CEGMA completeness (%) (complete/partial)	98.0/98.8	98.8/99.6
Avg CEG gene no. (complete/partial)	1.12/1.16	1.11/1.15
BUSCO eukaryote completeness (%) Complete and single-copy/Complete and duplicated	81.6/0.4	86.7/0.4

Table S2. DNA and RNA sequencing libraries and data

Species	Source	Sample type	Accession number	Library type	Sequencing platform	Read length (bp)	Used for
Bursaphelenchus xylophilus	*DNA*	male	DRX233522	Genomic pair-end	HiSeq 2000	151	Depth/SNP comparison
		male	DRX233523	Genomic pair-end	HiSeq 2000	101	Depth/SNP comparison
		male	DRX233524	Genomic pair-end	HiSeq 2000	151	Depth/SNP comparison
		female	DRX233525	Genomic pair-end	HiSeq 2000	151	Depth/SNP comparison
		female	DRX233526	Genomic pair-end	HiSeq 2000	101	Depth/SNP comparison
		female	DRX233527	Genomic pair-end	HiSeq 2000	151	Depth/SNP comparison
		Ka4C1 mixed stage	DRX061162	Genomic pair-end	HiSeq 2500	101	population comparion
		S10-P9 mixed stwage	DRX061165	Genomic pair-end	HiSeq 2500	101	population comparion
Bursaphelenchus okinawaensis	*DNA*	male	DRX233528	Genomic pair-end	HiSeq 2000	151	Depth/SNP comparison
		male	DRX233529	Genomic pair-end	HiSeq 2000	101	Depth/SNP comparison
		male	DRX233530	Genomic pair-end	HiSeq 2000	151	Depth/SNP comparison
		hermaphrodite	DRX233531	Genomic pair-end	HiSeq 2000	151	Depth/SNP comparison
		hermaphrodite	DRX233532	Genomic pair-end	HiSeq 2000	101	Depth/SNP comparison
		hermaphrodite	DRX233533	Genomic pair-end	HiSeq 2000	151	Depth/SNP comparison
		SH1 mixed state	DRX233491	Genomic pair-end	HiSeq 2000	101	population comparion
		SH3 mixed stage	DRX233517	Genomic pair-end	HiSeq 2000	151	population comparion
		sy867 male	DRX233519	Genomic single	HiSeq 2000	151	mutant genome seq
		sy867 hermaphrodite	DRX233518	Genomic single	HiSeq 2000	151	mutant genome seq
		sy868 male	DRX233521	Genomic single	HiSeq 2000	151	mutant genome seq
		sy868 hermaphrodite	DRX233520	Genomic single	HiSeq 2000	151	mutant genome seq
	RNA	male	DRX233502	polyA-paired	HiSeq 2000	151	*tra-1* RNAseq
		male	DRX233503	polyA-paired	HiSeq 2000	151	*tra-1* RNAseq
		male	DRX233504	polyA-paired	HiSeq 2000	151	*tra-1* RNAseq
		hermaphrodite	DRX233505	polyA-paired	HiSeq 2000	151	*tra-1* RNAseq
		hermaphrodite	DRX233506	polyA-paired	HiSeq 2000	151	*tra-1* RNAseq
		hermaphrodite	DRX233507	polyA-paired	HiSeq 2000	151	*tra-1* RNAseq
		male (sy867)	DRX233511	polyA-single	HiSeq 2000	101	*tra-1* RNAseq
		male (sy867)	DRX233512	polyA-single	HiSeq 2000	101	*tra-1* RNAseq
		male (sy867)	DRX233513	polyA-single	HiSeq 2000	101	*tra-1* RNAseq
		hermaphrodite (sy867)	DRX233514	polyA-single	HiSeq 2000	101	*tra-1* RNAseq
		hermaphrodite (sy867)	DRX233515	polyA-single	HiSeq 2000	101	*tra-1* RNAseq
		hermaphrodite (sy867)	DRX233516	polyA-single	HiSeq 2000	101	*tra-1* RNAseq

Table S3. Male specific contigs in *B. okinawaensis* identified by kmer-based comparison using the DiscoverY program

Contig name	Contig length	Female Propotion	Male normalised depth	Sequence
NODE_101389	79	0.00	13.50	AAAAAAAAAAAAAAAAAAAAAAAAAAAAAAAAAAAAAAAAAAAAAAAAAAAAAAAAAAAAAAAAAAAAAAAAAAAGGGG
NODE_101397	79	0.00	2.50	TTCCTTTTTTTTTTTTTTTTTTTTTTTTTTTTTTTTTTTTTTTTTTTTTTTTTTTTTTTTTTTTTTTTTTTTTTTTTTT
NODE_101525	78	0.00	5505.00	TTTTTTTTTTTTTTTTTTTTTTTTTTTTTTTTTTTTTTTTTTTTTTTTTTTTTTTTTTTTTTTTTTTTTTTTTTTTTT
NODE_102566	78	0.00	37.00	CTTTTTTTTTTTTTTTTTTTTTTTTTTTTTTTTTTTTTTTTTTTTTTTTTTTTTTTTTTTTTTTTTTTTTTTTTTTTT
NODE_102697	78	0.00	17.00	CCTTTTTTTTTTTTTTTTTTTTTTTTTTTTTTTTTTTTTTTTTTTTTTTTTTTTTTTTTTTTTTTTTTTTTTTTTTTT
NODE_96172	92	0.00	9.13	TTTTTTTTTTTTTTTCTTTTTTTTTTTTTTTTTTTTTTTTTTTTTTTTTTTTTTTTTTTTTTTTTTTTTTTTTTTTTTTTTTTTTTTTTTTT
NODE_102351	78	0.02	113.00	TTTTTTTTTTTTTTTTTTTTTTTTTTTTTTTTTTTTTTTTTTTTTTTTTTTTTTTTTTTTTTTTTTTTTTTTTTTTTG
NODE_90740	152	0.02	38.01	TTTTTTTTTTTTTTTTTTTTTTTTTTTTTTTTTTTTTTTTTTTTTTTTTTTTTTTTTTTTTTTTTTTTTTTTTTGTTTTTTTTTTTTTTTTTTTTTTTTTTTTTTTTTTTTTTTTTTTTTTTTTTTTTTTTTTTTTTTTTTTTTTTTTTTTTTT
NODE_102467	78	0.04	68.00	TTTTTTTTTTTTTTTTTTTTTTTTTTTTTTTTTTTTTTTTTTTTTTTTTTTTTTTTTTTTTTTTTTTTTTTTTTTTGT
NODE_101314	79	0.06	29.50	TTTTTTTTTTTTTTTTTTTTTTTTTTTTTTTTTTTTTTTTTTTTTTTTTTTTTTTTTTTTTTTTTTTTTTTTTTTTGGG
NODE_102491	78	0.06	61.00	TTTTTTTTTTTTTTTTTTTTTTTTTTTTTTTTTTTTTTTTTTTTTTTTTTTTTTTTTTTTTTTTTTTTTTTTTTTGTT
NODE_102723	78	0.06	9.00	TTTTTTTTTTTTTTTTTTTTTTTTTTTTTTTTTTTTTTTTTTTTTTTTTTTTTTTTTTTTTTTTTTTTTTTTTTTGTG
NODE_102652	78	0.08	21.00	TTTTTTTTTTTTTTTTTTTTTTTTTTTTTTTTTTTTTTTTTTTTTTTTTTTTTTTTTTTTTTTTTTTTTTTTTTGGGG
NODE_91835	137	0.16	0.75	TTTTTTTTTTTTTTTTTAAAAAAAATTTTTTTTTTTTTTTTTTTTTTAAAAAAATTTTTTTTTTTTTTTTTTTTTTAAAAAAAATTTTTTTTTTTTTTTTTTTTTTAAAAAAATTTTTTTTTTTTTTTTTTTTTT
NODE_96905	89	0.27	20.25	TTTTTTTTTTTTTTTTTTTTTTTTTTTTTTTTTTTTTTTTTTTTTTTTTTTTTTTTTTTTTTTTTTTTTTTTTGGGGGGGGGGGGGGGG
NODE_93952	107	0.32	1.33	TTTTTTTTTTTTTAAAAAAAATTTTTTTCCTTTTTTTTTTTTTAAAAAAAATTTTTTTCCTTTTTTTTTTTTTAAAAAAAATTTTTTTCCTTTTTTTTTTTTTAAAA
NODE_101386	79	0.37	16.50	TTTTTTTTTTTTTTTTTTTTTTTTTTTTTTTTTTTTTTTTTTTTTTTTTTTTTTTTTTTTTTGGGGGGGGGGGGGGGGGG
NODE_102689	78	0.40	18.00	TTTTTTTTTTTTTTTTTTTTTTTTTTTTTTTTTTTTTTTTTTTTTTTTTTTTTTTTTTTTGGGGGGGGGGGGGGGGGGG
NODE_68210	248	0.53	0.76	TGAATCTTCTTTAGATTTCTGCACCATGTGAAACCCACTTTTACTTCTTTTTTTCTTCTTTTTTTCTTTTTTTTTTTTTTTTTTTTCTTTTTTTTTTTTTTTTTTTTTTTTTTTTTTTTTCCCTTTTTTTTTTTTTTTTTTTTCTTTTCTTTTTTTTTTTTTTTTTTTTTTTTTTTTTTTTTTTTTTTTTTTTGGGGGTTTTTTTTTTTTTTTTTTTATTTTAATGTTTTTTTGGGGTTTTTTTGTTTTTTTATTAA
NODE_93945	107	0.61	3.70	ATTTTTTTTTTTTTAAATAAATTTCTTTCCATTTTTTTTTTTTTAAATAAATTTCTTTCCATTTTTTTTTTTTTAAATAAATTTCTTTCCATTTTTTTTTTTTTAAA
NODE_88421	229	0.62	0.86	TTGTTTTTTTTGTTTTGTATAAAAAGTTTTATCATTTTTTTTTTTTTTTTTAATTTTTTTTGTATTTTTATTTATTTTTTTTATTTTTATTTGTTTTTTTTTTTTTTTTTTTTTTAATTGTTTTTTTTTTTTTTTTTTTTTTTTTTTTTATTTTTTTTTTTTTTTTGTGTTTTTTTTTTTTTTTTTTTTTGTGGGGGTTTTTTTTTTTTTTTTTGGGGGGGGGGGAAAA
NODE_81197	235	0.63	42.26	TAGGGTGGGGTAGGGTAGGGTAGGGTAGGGTAGGGTAGGATAGGGTAGGGTAGGGTAGGGTAGGGTAGGGTAGGGTACGGAACGGTACGGTACAGTAGGATAGGGTAGTGGTGGATAGGGTAGGGTAGTATATTTGTTTTTTTGCAGTGCAGTGTCCTTTTTTTTTTTTTTTCTTTTTTTTTTTTTTTTTTTTTTTTTTTTTTTTTTTTTTTTTTTTTTTTTTTTTTTTTTTTTTTTTTTTTT
NODE_99382	82	0.65	23.42	TTGAAAAATCGGTCCACAGCAGGGACATCGATTTTCCAAAAAATTTTTTTTTGGTTTTTTTGCGTACAGGCACCACCTAC
NODE_95637	94	0.72	338.76	AAAAAAATTAAAGTTTTCAAAAAAACCATCAAAATTTTGAAAAATTTGAAATTTCTCGAAAAAAAGTCACTATGTAACAAACTTTTGACAACAG
NODE_98012	85	0.73	37.25	ACCAATCAAAAATCACAAAAAATTTTTTGTTTCTAACACCTTTTTAAAAAAAGTTATGCTTGAAAAAAATAGGTTGCCTGTTTTT
NODE_102283	78	0.73	146.00	TTTGCGGATCTGCGGTTTTTAGACACTTGCGGATCTTCGGTTTTTGGACACTTGCGGATTTTTTAAAGGTTTTTTTTT
NODE_99073	82	0.73	323.60	AGTTTTTGCCATTTTTTGTTTTGTAAAGAAAGTTTTTGCCATTTTTTGTTTTGTAAAGAAAGTTTTTGCCATTTTTTGTTTT
NODE_100828	79	0.73	841.00	AATAAAAAAATAAAAAAAACCCCTTTAAAAAATCCGCAAATGTCCAAAATCCGCAAGTGTCCAAAAACCGAAGATCCGCA
NODE_98605	83	0.74	277.17	ACACTTGCGGATCTTCGGTTTTTGGACACTTGCGGATTTTGGACATTTGCGGATTTTTTAAAGGGGTTTTTTTTTATTTTTTA
NODE_100489	80	0.74	32.67	TTAAAAAAACTGTTTTAAAAAATTGCAAATAAAACATATTTTTAGGAGTTTTTAAGCAAAAAGAAAGCAGTTTTAGCGCTC
NODE_96987	88	0.74	427.55	GCAATTAAGGTTTTTAAACTTCGACGTAATAAAATTTTTTAAAAAACCCCCACGAGGGGTAAATTTTTTTTCGGTTTTTGACTAATTT
NODE_93765	108	0.74	157.55	AAAACATATTTTTAGGAGTTTTTAAGCAAAAAGAAAGCAGTTTTAGCGCTCCTTGCTTTTTTTAAAAATTAAAAAAAAATTTTTTGCGGACCGGACCGGACCCAAAAA
NODE_89683	207	0.75	2.32	AAAAAAAAAAAAAAAAAAAAAAAAAAAAAAAAAAAAGAAAAAAAAAAAAAAAAAAAAAAAAGTAGTGGGGGGGGGGGGGGGGGGGGGGGGGGGGGGGGGGGGGGGGGGGGGGGGGGGGGGGAGGAAATCAGGGCTACCTGTGGACCAAAAAATTTTTTTGGGTCTCCACCCCCCCCCCCTCCCAGAAAAAATCCGTAGCGACGCCCCTG
NODE_95196	97	0.75	64.60	CTGCGGTTTTTAGACACTTTCGGATCTTCGGTTTTTGGACACTTGCGGATTTTGGACATTTGCGGATTTTTTAAAGGTTTTTTTTTATTTTTTAGTT

Table S4. Primer list for chromosome mapping in *B. xylophilus* .

Primer set No.	Description	Primer sequence 5'-3'	Chr	Position
I-A	BxyChrI_409323_F	CTGAAGTTCGACACTCAGCG	Chr1	2346740
	BxyChrI_409323_R	CACCGCATCCAAGAAGATGG		2345588
I-B	BxyChrI_83786_F	AAACGAAACGACCGGTCTTG	Chr1	1253005
	BxyChrI_83786_R	CAAGGTTAGCGAATCGGACG		1251971
I-C	BxyChrI_678105_F	GCATTCTCATGTCCGAGCAG	Chr1	4324510
	BxyChrI_678105_R	ATAATTTCGGAGGCCAACGC		4325434
I-D	BxyChrI_591146_F	TTCATTAAACGCCCACAGCC	Chr1	5456099
	BxyChrI_591146_R	CCCAATCAAGGCCAATGGAG		5455025
I-E	BxyChrI_218107_F	TGTGGTTGAGCGTGTTTAGC	Chr1	14713315
	BxyChrI_218107_R	GAGGTCTGTGTGCAGTTCAC		14712163
II-A	BxyChrII_3253994_F	CAAAGTGAGATCGAGGCACG	Chr2	11566467
	BxyChrII_3253994_R	GATCATTCTCGGCGTGATCG		11567467
II-B	BxyChrII_1433720_F	GACGTCCTCGACAATTCGTG	Chr2	3067845
	BxyChrII_1433720_R	ATTGGCGACTTATGACGTGC		3066696
II-C	BxyChrII_237779_F	ACCGATGGATTGACCTAGCC	Chr2	1958903
	BxyChrII_237779_R	ATTGGACCAAAGTTCCGTGC		1957739
II-D	BxyChrII_1388801_F	GGTCATCAGCGTTTGGTCTC	Chr2	7092499
	BxyChrII_1388801_R	ATCTCGGAAGTGACCAGTCC		7091369
II-E	BxyChrII_625388_F	TTCCAGGAGTTAGGTGAGCG	Chr2	4732834
	BxyChrII_625388_R	ATCGCGCTGAAATTTAGGGC		4731780
III-A	BxyChrIII_2747166_F	CGTCTTCGCATCTGTTCCAG	Chr3	225750
	BxyChrIII_2747166_R	GAACCAGTCGATCGAAAGGC		224749
III-B	BxyChrIII_258368_F	TTTAGCCCGCGATTACCAGG	Chr3	9942256
	BxyChrIII_258368_R	ATGACCACTCACAGCTCACC		9941105
III-C	BxyChrIII_268247_F	TAGGGATCGACACAAGGCAG	Chr3	4472112
	BxyChrIII_268247_R	GAAGGAAGCAATGGCCTCTG		4473204
III-D	BxyChrIII_567570_F	GTGAACGGCCTCAAGACAAG	Chr3	3276843
	BxyChrIII_567570_R	TCACGTTTCGAACTCCTTGC		3275921
III-E	BxyChrIII_280579_F	TTCGAAACACAAGGATCCGC	Chr3	6160408
	BxyChrIII_280579_R	GAACACGATGGCGAAGGATC		6159454
IV-A	BxyChrIV_4055327_F	AGCGGATAGACCACTTCTCG	Chr4	12719916
	BxyChrIV_4055327_R	TGCTGAGTGATGTGGAGAGG		12720916
IV-B	BxyChrIV_1395981_F	AGAAATCAGCGACAACTGCC	Chr4	4486449
	BxyChrIV_1395981_R	TGTAAGCCGTTGGTTATGCG		4485407
IV-C	BxyChrIV_1761249_F	GATGGAATCGTCGGACTTGC	Chr4	3311499
	BxyChrIV_1761249_R	AATTGCACCAGGTCTGCATG		3312460
IV-D	BxyChrIV_449397_F	GAGCCCACTCCAAACGAATC	Chr4	322359
	BxyChrIV_449397_R	GAAGCCGTCCATGTCCATTC		323504
IV-E	BxyChrIV_164376_F	ACATGCGGAATTCATGACCG	Chr4	6458623
	BxyChrIV_164376_R	GAGCATGGATCTCGCAAAGG		6459777
V-A	BxyChrV_1724050_F	TCCATCGTTGGTTCCTCTCC	Chr5	10185655
	BxyChrV_1724050_R	CTTTGCGCGCATGAAATACG		10186746
V-B	BxyChrV_1030032_F	ATGCACTTTGCCCACATGTC	Chr5	3291089
	BxyChrV_1030032_R	CCTAAGCGTTCAATCCAGCC		3290154
V-C	BxyChrV_456531_F	GACAGCGAAGAGGAGGAGAG	Chr5	2000978
	BxyChrV_456531_R	CCATTTCCGAATCCGTGTCC		2001992
V-D	BxyChrV_214696_F	AGTCGTCAGAGAGCATGAGG	Chr5	741844
	BxyChrV_214696_R	AACACATTACACCGGCTTCG		740915
V-E	BxyChrV_510250_F	AGGAAAGCTCACGGAGGATC	Chr5	1018231
	BxyChrV_510250_R	ACGGAAATGAGGATGTTGCG		1017473
X-A	BxyChrX_1519028_F	CAGATGTTGACGAGCATGGG	Chr6	749002
	BxyChrX_1519028_R	ATTGCGACGAATCCATCTGC		747751
X-B	BxyChrX_1613213_F	ACTCCAGGGAGCGATTATCG	Chr6	653919
	BxyChrX_1613213_R	TACACAACGCTCGCTTCTTG		652773
X-C	BxyChrX_1142235_F	TCCAAGTGCCGCATTAAGAC	Chr6	9013567
	BxyChrX_1142235_R	GATTGGTGGTGGTTTGGAGG		9014701
X-D	BxyChrX_741686_F	CCGACCGCATGATGGATTAC	Chr6	10352738
	BxyChrX_741686_R	TCAACTTTCCCTGCACAGTG		10353920
X-E	BxyChrX_504167_F	ACCAGACAGACTGCAGAGTG	Chr6	12708840
	BxyChrX_504167_R	GGTGCAGTCGACAAATCTCC		12709966

Table S5. Effect of environmental stimuli and developmental history on male ratio in *B. okinawaensis* SH1 strain.

Treatments or developmental life history	Total			Individual
	No. herm	No. male	Percent male (%)	mean (%) ± SD
25°C incubation, 7day	2464	12	0.49	0.49 ± 0.74
20°C incubation, 7day	1448	10	0.69	0.66 ± 0.86
30°C incubation, 7day	1882	32	1.70	1.98 ± 2.23
25°C incubation, 14day	1803	7	0.39	0.60 ± 0.66
20°C incubation, 14day	1385	13	0.93	0.89 ± 0.88
30°C incubation, 14day	2208	20	0.91	0.86 ± 0.74
High population density	9090	47	0.51	0.52 ± 0.32
Heat shock (40°C, 2h)	3318	24	0.72	0.72 ± 0.60
Heat shock (40°C, 4h)	1972	28	1.40	1.72 ± 1.66
Ethanol exposure (10%, 1h)	2645	4	0.15	0.15 ± 0.20
After dauer recovery (P0)	125	0	0.00	0.00 ± 0.00
After dauer recovery (F1)	2639	0	0.00	0.00 ± 0.00
After dauer recovery (F2)	1642	13	0.79	0.74 ± 0.60
L4 starvation	1861	17	0.91	1.00 ± 1.45

Table S6. Primer list used for bacteria detection.

Name	Primer sequence 5'-3'	Reference
8F	AGAGTTTGATCCTGGCTCAG	Turner et al.[1]
1391R	GACGGGCGGTGTGTRCA	Turner et al.[1]
27F	AGAGTTTGATCCTGGCTCAG	Lane[2]
1492R	GGGTTACCTTGTTACGACTT	Lane[2]

Table S7. Primer list for chromosome mapping in *B. okinawaensis* .

Primer set No.	Description	Primer sequence 5'-3'	Chr	Position
I-A	BokChrI_467303_F	CGAGGACAAAGACGGTGAAC	Chr1	516299
	BokChrI_467303_R	CGTTAAACCGACCGCTATGG		517053
I-B	BokChrI_217459_F	CACTGAATCCGCTCATTGGG	Chr1	2674753
	BokChI_217459_R	CAACACGCGCTTTGAAACAG		2673837
II-A	BokChrII_145061_F	TATTGGCAGCGACCCATTTC	Chr2	6672087
	BokChrII_145061_R	TCCATCCAGTTATCGACCGG		6671315
II-B	BokChrII_28007_F	AAGCCTAGCCTAAGCCTAGC	Chr2	9706667
	BokChrII_28007_R	AGCTTTGAGAAAGAGCGCAG		9705734
III-A	BokChrIII_557955_F	TTTGCTTTGAGAGGAAGCCC	Chr3	3706198
	BokChrIII_557955_R	GGATGGAGGAAGACCCGTAG		3705174
III-B	BokChrIII_208599_F	ATGTTTGGCTGCAACTGGAG	Chr3	7688404
	BokChrIII_208599_R	TTCCGTTCCATCGACTTTGC		7687573
IV-A	BokChrIV_649768_F	GCTCTGTGCACACTAATCCG	Chr4	9892424
	BokChrIV_649768_R	CCGTCTTTGATGAGTGTGGC		9891211
IV-B	BokChrIV_354850_F	CGTGCACCTTTGATTCAACG	Chr4	9328161
	BokChrIV_354850_R	GTGGAATGCTGGACGCTAAG		9329225
V-A	BokChrV_48386_F	GGATTACTACGGCACTGCAC	Chr5	9579812
	BokChrV_48386_R	TTTCGTCCGTAATGCCACAC		9580651
V-B	BokChrV_73783_F	TCTGGGTGGAAACGGAGATC	Chr5	3804267
	BokChrV_73783_R	ATTCACTGATCGCACACTGC		3805384
X-A	BokChrX_166395_F	CTAGCTTGACGCAGCACTTC	Chr6	8697529
	BokChrX_166395_R	CAAACTTGCGACCAAATGGC		8696497
X-B	BokChrX_76368_F	TGTGTTGACTGTTGTCGCAG	Chr6	681041
	BokChrX_76368_R	ATATTCGCTGCTGGAAAGCC		682025

Table S8. Primer list for assigning linkage of a mutation to three regions (reg0008 on chr2, 0023 on chr3, and 0111 on chr5).

Primer set No.	Description	Primer sequence 5'-3'	Chromosome region	Position
34015	Bok_34015_F	TAACCTAGCGTAGTCAGGCG	reg0008 (chr2; 441K-1841K)	475481
	Bok_34015_R	TCAGACCGGACCTGGATTTG		476241
569774	Bok_569774_F	AACATTGGCCTGGGTTGTTC	reg0008 (chr2; 441K-1841K)	1019146
	Bok_569774_R	AGGTATGGAGTTGGGCCTTC		1020318
87440	Bok_87440_F	GGCCCAAAGGTTCTTCTTGG	reg0111 (chr5; 5760K-5909K)	5820273
	Bok_87440_R	TGTCACGACATGCAACTTCC		5819197
114208	Bok_114208_F	GCCGGATCTCTTACGTTTGG	reg0111 (chr5; 5760K-5909K)	5789346
	Bok_114208_R	AACTACCTCGGCTTCTGGAC		5787825
254138	Bok_254138_F	TTGAAGATCGCAATCAGCCG	reg0023 (chr3; 5145K-5995K)	5398493
	Bok_254138_R	CGATCAAGATGTCGGCTTGG		5399351
386368	Bok_386368_F	AGACTTTGTCGCTGCCAATG	reg0023 (chr3; 5145K-5995K)	5530705
	Bok_386368_R	TGGTTGTGTTCAAGGCATCG		5531757
574153	Bok_574153_F	GTCTGCATCGGCCCATAAAG	reg0023 (chr3; 5145K-5995K)	5718606
	Bok_574153_R	CAGAATCCGACGAAAGCGAG		5719617

Table S9. Primer list for *tra-1* sequencing.

Primer name	Primer sequence 5'-3'
tra-1-F1	AATGCTTGTGACCCCACCTG
tra-1-R1	TGGATGCGGATTCTGGAGTG
tra-1-F2(509)	CCCTCGACCCAACTGTTCTC
tra-1-F3(1084)	AAGAACGACGGTAAGCCTGG
tra-1-F4(1606)	AACAGTACTGGTGCCGATGG
tra-1-F5(2204)	AAAGCTACACAGACCCCAGC
tra-1-F6(2776)	GGACGACCCTGAACTGGAAG

Supplementary references

1. Turner, S., Pryer, K. M., Miao, V. P. & Palmer, J. D. Investigating deep phylogenetic relationships among cyanobacteria and plastids by small subunit rRNA sequence analysis. *J. Eukaryot. Microbiol*. 46, 327-338 (1999)

2. Lane, D. J. 16S/23S rRNA sequencing. *Nucleic acid techniques in bacterial systematics*. pp. 115-175 (John Wiley & Sons, New York, 1991).
