# Supplementary material for: Possible stochastic sex determination in Bursaphelenchus nematodes
Source: Nat Commun. 2022 May 11;13:2574. doi: 10.1038/s41467-022-30173-2 (PMC9095866; doi:10.1038/s41467-022-30173-2)
Supplement: Supplementary file 2 — Description of Additional Supplementary Files [file 41467_2022_30173_MOESM2_ESM.pdf]

Description of Additional Supplementary Files

File Name: Supplementary Data 1
Description: Differentially expressed genes in *B. okinawaensis* wild-type hermaphrodites relative to wild-type males. Genes upregulated in the hermaphrodites or males are defined as feminine genes (highlighted in red) and masculine genes (highlighted in blue), respectively.

File Name: Supplementary Data 2
Description: Significantly enriched GO terms for genes that were differentially expressed (DEs) between the wild-type females and males.

File Name: Supplementary Data 3
Description: Differentially expressed genes in *B. okinawaensis sy867* hermaphrodites (+/+, *tra*/+) relative to wild-type hermaphrodites. Feminine genes and masculine genes are highlighted in red and blue, respectively.

File Name: Supplementary Data 4
Description: Significantly enriched GO terms for genes that were differentially expressed (DEs) between the wild-type hermaphrodite and tra-mutant (*sy867*) hermaphrodite (+/+, *tra*/+).

File Name: Supplementary Data 5
Description: Differentially expressed genes in *B. okinawaensis sy867* males (*tra*/*tra*) relative to wild-type males. Feminine genes and masculine genes are highlighted in red and blue, respectively.

File Name: Supplementary Data 6
Description: Significantly enriched GO terms for genes that were differentially expressed (DEs) between the wild-type male and tra-mutant (*sy867*) male (*tra*/*tra*).

File Name: Supplementary Data 7
Description: Cluster 2 gene list in Figure 6d.

File Name: Supplementary Data 8
Description: Cluster 5 gene list in Figure 6d.
