# Supplementary material for: Possible stochastic sex determination in Bursaphelenchus nematodes
Source: Nat Commun. 2022 May 11;13:2574. doi: 10.1038/s41467-022-30173-2 (PMC9095866; doi:10.1038/s41467-022-30173-2)
Supplement: Supplementary file 11 — Reporting Summary [file 41467_2022_30173_MOESM11_ESM.pdf]

# nature portfolio

Corresponding author(s): Paul W Sternberg
Taisei Kikuchi

Last updated by author(s): Apr 19, 2022

# Reporting Summary

Nature Portfolio wishes to improve the reproducibility of the work that we publish. This form provides structure for consistency and transparency in reporting. For further information on Nature Portfolio policies, see our Editorial Policies and the Editorial Policy Checklist.

## Statistics

For all statistical analyses, confirm that the following items are present in the figure legend, table legend, main text, or Methods section.

| n/a | Confirmed | |
|---|---|---|
| ☐ | ☒ | The exact sample size (*n*) for each experimental group/condition, given as a discrete number and unit of measurement |
| ☐ | ☒ | A statement on whether measurements were taken from distinct samples or whether the same sample was measured repeatedly |
| ☐ | ☒ | The statistical test(s) used AND whether they are one- or two-sided<br>*Only common tests should be described solely by name; describe more complex techniques in the Methods section.* |
| ☒ | ☐ | A description of all covariates tested |
| ☒ | ☐ | A description of any assumptions or corrections, such as tests of normality and adjustment for multiple comparisons |
| ☒ | ☐ | A full description of the statistical parameters including central tendency (e.g. means) or other basic estimates (e.g. regression coefficient) AND variation (e.g. standard deviation) or associated estimates of uncertainty (e.g. confidence intervals) |
| ☐ | ☒ | For null hypothesis testing, the test statistic (e.g. *F*, *t*, *r*) with confidence intervals, effect sizes, degrees of freedom and *P* value noted<br>*Give P values as exact values whenever suitable.* |
| ☒ | ☐ | For Bayesian analysis, information on the choice of priors and Markov chain Monte Carlo settings |
| ☒ | ☐ | For hierarchical and complex designs, identification of the appropriate level for tests and full reporting of outcomes |
| ☒ | ☐ | Estimates of effect sizes (e.g. Cohen's *d*, Pearson's *r*), indicating how they were calculated |

*Our web collection on statistics for biologists contains articles on many of the points above.*

## Software and code

Policy information about availability of computer code

| | |
|---|---|
| Data collection | No software was used |
| Data analysis | programs used for sequence analysis are state and referenced in the manuscript as follows: Cufflinks packages v2.2.1<br>EdgeR v3.2.4<br>HTSeq v0.9.1<br>Hisat2 v2.1.0<br>find_CAPS.py v2012<br>K-mer Counter ver.3.1.0<br>DiscoverY v2019<br>icorn2 version 2<br>Gapfiller (v2.1.2)<br>Image (v2.4.1)<br>design_primers.v3.py<br>Genome Analysis Toolkit (ver. 3.3.0) SMALT v0.7.4<br>K-mer Counter DiscoverY vcftools v0.1.13<br>OrthoFinder (v0.2.8) Augustus (v. 3.0.1 ICORN2<br>MaSuRCa assembler v2.2.1<br>RTA 1.12.4.2<br>Smudgeplot v0.2.5<br>Flye (v2.8.3-b1695)<br>3D-DNA pipe-line (v201008)<br>Augustus (v.3.0.1) |

OrthoFinder (v.0.2.8)
Mosdepth v0.2.9
CNVkit v0.9.9
Genome Analysis Toolkit (ver.3.3.8)
vcftools v0.1.13
EdgeR v3.2.4

For manuscripts utilizing custom algorithms or software that are central to the research but not yet described in published literature, software must be made available to editors and reviewers. We strongly encourage code deposition in a community repository (e.g. GitHub). See the Nature Portfolio guidelines for submitting code & software for further information.

## Data

Policy information about availability of data

All manuscripts must include a data availability statement. This statement should provide the following information, where applicable:

- Accession codes, unique identifiers, or web links for publicly available datasets
- A description of any restrictions on data availability
- For clinical datasets or third party data, please ensure that the statement adheres to our policy

Data Availability
All sequence data generated during and/or analysed during the current study have been deposited at DDBJ/ENA/GenBank under BioProject accession PRJEB40022 (https://identifiers.org/resolve?query=ena.embl:PRJEB40022), PRJEB40023 (https://identifiers.org/resolve?query=ena.embl:PRJEB40023) and PRJDB10466 (https://identifiers.org/resolve?query=ena.embl:PRJDB10466). All other relevant data are available from the authors.

All sequence data generated during and/or analysed during the current study have been deposited at DDBJ/ENA/GenBank under BioProject accession PRJEB40022 (https://identifiers.org/resolve?query=ena.embl:PRJEB40022), PRJEB40023 (https://identifiers.org/resolve?query=ena.embl:PRJEB40023) and PRJDB10466 (https://identifiers.org/resolve?query=ena.embl:PRJDB10466) The reference genomes, v5.1 for B. xylophilus and v2.0 for B. okinawaensis are available from GenBank assembly accessions GCA_904066235.2 and GCA_904066225.2, respectively. All other relevant data are available from the authors

# Field-specific reporting

Please select the one below that is the best fit for your research. If you are not sure, read the appropriate sections before making your selection.

☒ Life sciences ☐ Behavioural & social sciences ☐ Ecological, evolutionary & environmental sciences

For a reference copy of the document with all sections, see nature.com/documents/nr-reporting-summary-flat.pdf

# Life sciences study design

All studies must disclose on these points even when the disclosure is negative.

| | |
|---|---|
| Sample size | sample sizes were chosen based on experience with these simple experiments . Sample sizes were chosen based on our experience and many published works. |
| Data exclusions | no exclusions |
| Replication | independent replicates for relevant experiments, all experiments had at least three independent replications. |
| Randomization | worms picked at random with platinum wires. The organisms were randomly allocated. |
| Blinding | Blinding not necessary given the phenotypes scored are visually-based and obvious. Blinding was not relevant to this study. |

# Reporting for specific materials, systems and methods

We require information from authors about some types of materials, experimental systems and methods used in many studies. Here, indicate whether each material, system or method listed is relevant to your study. If you are not sure if a list item applies to your research, read the appropriate section before selecting a response.

## Materials & experimental systems

| n/a | Involved in the study |
|---|---|
| ☒ | ☐ Antibodies |
| ☒ | ☐ Eukaryotic cell lines |
| ☒ | ☐ Palaeontology and archaeology |
| ☐ | ☒ Animals and other organisms |
| ☒ | ☐ Human research participants |
| ☒ | ☐ Clinical data |
| ☒ | ☐ Dual use research of concern |

## Methods

| n/a | Involved in the study |
|---|---|
| ☒ | ☐ ChIP-seq |
| ☒ | ☐ Flow cytometry |
| ☒ | ☐ MRI-based neuroimaging |

# Animals and other organisms

Policy information about studies involving animals; ARRIVE guidelines recommended for reporting animal research

| | |
|---|---|
| Laboratory animals | Bursaphelenchus xylophilus,  Ka4C1 and S10-P9 strain<br>Bursaphelenchus okinawaensis, SH1, SH3 strain |
| Wild animals | not applicable |
| Field-collected samples | not applicable: nematode strains previously described |
| Ethics oversight | no ethical guidance needed for nematode studies. |

Note that full information on the approval of the study protocol must also be provided in the manuscript.
